# Evidence for Effects of Extracellular Vesicles on Physical, Inflammatory, Transcriptome and Reward Behaviour Status in Mice

**DOI:** 10.3390/ijms23031028

**Published:** 2022-01-18

**Authors:** Nagiua Cuomo-Haymour, Hannes Sigrist, Christian Ineichen, Giancarlo Russo, Ursina Nüesch, Felix Gantenbein, Luka Kulic, Irene Knuesel, Giorgio Bergamini, Christopher Robert Pryce

**Affiliations:** 1 Preclinical Laboratory for Translational Research into Affective Disorders, Department of Psychiatry, Psychotherapy and Psychosomatics Psychiatric Hospital, University of Zurich, 8008 Zurich, Switzerland; nagiua.haymour@bli.uzh.ch (N.C.-H.); hannes.sigrist@bli.uzh.ch (H.S.); christian.ineichen@usz.ch (C.I.); bergamini.giorgio@gmail.com (G.B.); 2 Neuroscience Center Zurich, 8057 Zurich, Switzerland; 3 Functional Genomics Centre Zurich, University of Zurich and Swiss Federal Institute of Technology Zurich, 8057 Zurich, Switzerland; giancarlo1russo@gmail.com; 4 Paediatric Immunology, University Children’s Hospital Zurich, 8032 Zurich, Switzerland; ursina.nueesch@kispi.uzh.ch; 5 Zurich Integrative Rodent Physiology, University of Zurich, 8057 Zurich, Switzerland; felix.gantenbein@uzh.ch; 6 Roche Pharmaceutical Research and Early Development, Roche Innovation Center Basel, 4070 Basel, Switzerland; luka.kulic@roche.com (L.K.); knuesel@me.com (I.K.)

**Keywords:** inflammation, extracellular vesicles, microRNA, lipopolysaccharide, chronic social stress, nucleus accumbens, reward

## Abstract

Immune-inflammatory activation impacts extracellular vesicles (EVs), including their miRNA cargo. There is evidence for changes in the EV miRNome in inflammation-associated neuropsychiatric disorders. This mouse study investigated: (1) effects of systemic lipopolysaccharide (LPS) and chronic social stress (CSS) on plasma EV miRNome; and (2) physiological, transcriptional, and behavioural effects of peripheral or central delivered LPS-activated EVs in recipient mice. LPS or CSS effects on the plasma EV miRNome were assessed by using microRNA sequencing. Recipient mice received plasma EVs isolated from LPS-treated or SAL-treated donor mice or vehicle only, either intravenously or into the nucleus accumbens (NAc), on three consecutive days. Bodyweight, spleen or NAc transcriptome and reward (sucrose) motivation were assessed. LPS and CSS increased the expression of 122 and decreased expression of 20 plasma EV miRNAs, respectively. Peripheral LPS-EVs reduced bodyweight, and both LPS-EVs and SAL-EVs increased spleen expression of immune-relevant genes. NAc-infused LPS-EVs increased the expression of 10 immune-inflammatory genes. Whereas motivation increased similarly across test days in all groups, the effect of test days was more pronounced in mice that received peripheral or central LPS-EVs compared with other groups. This study provides causal evidence that increased EV levels impact physiological and behavioural processes and are of potential relevance to neuropsychiatric disorders.

## 1. Introduction

Extracellular vesicles (EVs) are heterogeneous membrane-enclosed structures secreted by virtually all cell types, but most abundantly by immune cells [1], in the periphery and central nervous system (CNS) [2,3]. Various molecules are encapsulated into nascent EVs, including proteins, lipids, mRNAs, and high amounts of small non-coding RNAs, including microRNAs (miRNAs), thereby forming the EV “cargo” [3]. Loading of these molecules into EVs is a regulated process and dependent on the status of the parent cell [4]. Loaded EVs can be transported in body fluids over short and long distances and their functionally active cargo can be transferred to recipient cells [5,6,7,8]. As such, they constitute an important mechanism of cell-to-cell and organ-to-organ communication [9]. Evidence has been obtained for the integral involvement of EVs in the immune response including inflammation. The latter is associated with changes in number [10], periphery-CNS passage [11], and cargo [12] of peripheral and CNS EVs. In turn, immune-activated EVs potentiate downstream peripheral and central inflammatory responses by increasing expression and transport of pro-inflammatory molecules, including miRNAs, to and within CNS [13]. miRNAs are non-coding RNAs of 18–25 nucleotides that exert complex post-transcriptional regulation, primarily via mRNA degradation or translational repression [14]. EV miRNAs exert major regulatory functions in multiple immune-inflammatory processes [15] and are abundant and stable [16,17]. These findings suggest a potential aetio-pathophysiological role of EVs in immune system-associated CNS disorders, including autoimmune disorders such as multiple sclerosis (MS) [18] and neuropsychiatric disorders such as major depressive disorder (MDD) [19]. Indeed, recent studies have reported changes in the peripheral EV miRNA profile of patients with MS [20,21] or MDD [22] compared to healthy control subjects.

Therefore, whether or not EVs contribute to the aetio-pathophysiology of these brain disorders is certainly worthy of investigation and requires experimental animal studies. Some highly relevant evidence has already been obtained. In mice, inflammation-induced transfer of functional mRNA from EVs derived from peripheral hematopoietic cells to neurons, with a subsequent alteration in the latter’s miRNA profile, has been reported [8], thereby demonstrating that EVs—or at least their RNAs—can cross the blood–brain barrier (BBB), enter the brain parenchyma, and affect transcription in recipient neurons. Inflammation-induced transfer of astrocyte EVs into the peripheral circulation and a subsequent increase in acute peripheral cytokine response has also been shown in mice [23], indicating bidirectional EV transport and action across the BBB. Manipulations used to study acute or chronic inflammatory processes in rodents, including inflammation-associated brain-behavioural dysfunctions, include systemic administration of lipopolysaccharide (LPS) [24] and exposure to chronic social stress (CSS) [25,26], respectively. Dysregulation of specific EV miRNAs occurs both after LPS challenge [27] and chronic stress [28,29]. A small number of studies have investigated the physiological effects of exogenous, immune-activated EVs: Isolation of serum EVs from LPS-challenged donor mice and their peripheral administration to naïve recipients increased neuro-inflammation and peripheral and brain cytokine levels [27]. Peripheral infusion of plasma EVs isolated from donor rats challenged with interferon-β (IFN-β)- a major LPS-induced cytokine- increased expression of liver pro-inflammatory markers in naïve recipients [30]. The in vitro microglia response to LPS increased after the addition of serum EVs isolated from mice susceptible to chronic social defeat [31]. Alongside its activation of immune-inflammatory processes, LPS also induces “sickness behaviour” in rodents, including fatigue and reduced motivation for gustatory reward [32], and similar behavioural states are induced by CSS [25,33,34]. With respect to a causal role of EVs in such dysfunctions, very little is known to date. Evidence includes reduced locomotion in rats infused with plasma EVs isolated from IFN-β-challenged donors [30]. Studies investigating the potential pathophysiological role of EVs in processes of reward motivation are lacking. These would be important given that, for example, MS and MDD are often comorbid [35] and both can present attenuated reward processing mediated in part by changes in the mesocorticolimbic dopamine system including the nucleus accumbens (NAc) [36,37].

The overall aim of the present study was to investigate the effects of inflammatory challenges on peripheral circulating EVs in mice and then some selected effects of EVs derived from challenged donor mice in recipient mice. Firstly, we studied the effects of systemic LPS or CSS on the plasma EV miRNome. Secondly, we studied the effects of repeated intravenous injection of plasma/lymph node EVs from LPS-challenged or saline-challenged donor mice on physical status, peripheral inflammation markers, the spleen transcriptome, and effortful reward motivation in recipient mice. Third, we investigated the effects of repeated infusion of plasma EVs from LPS-challenged or saline-challenged donor mice onto the NAc in naïve recipient mice on their physical status, effortful reward motivation, and NAc transcriptome.

## 2. Results

### 2.1. Experiment 1: LPS and CSS Induce Changes in the Plasma EV miRNome

A pilot study was conducted into the effects of acute systemic LPS (*n* = 9) versus SAL (*n* = 7) on selected plasma EV miRNAs at 5 h post-injection using RT-qPCR. Seven miRNAs were tested—miR-146a-5p, miR-155-5p, miR-122-5p, miR-15a-5p, miR-16-5p, miR-26b-5p, and miR-132-5p. For five, expression levels were upregulated in LPS mice compared with SAL mice: namely, miR-146a-5p (*p* = 0.0006), miR-122-5p (*p* = 0.009), miR-155-5p (*p* = 0.04), miR-16-5p (*p* = 0.04), and miR-15a-5p (*p* = 0.03) (*p* values obtained with unpaired Student’s *t*-tests); on the other hand, there was no group effect for the other two miRNAs. This time interval between LPS administration and EV collection was then used for the Experiment 1 main study, as well as for blood collection from donor mice in Experiment 2 and Experiment 3.

**Figure 1:**
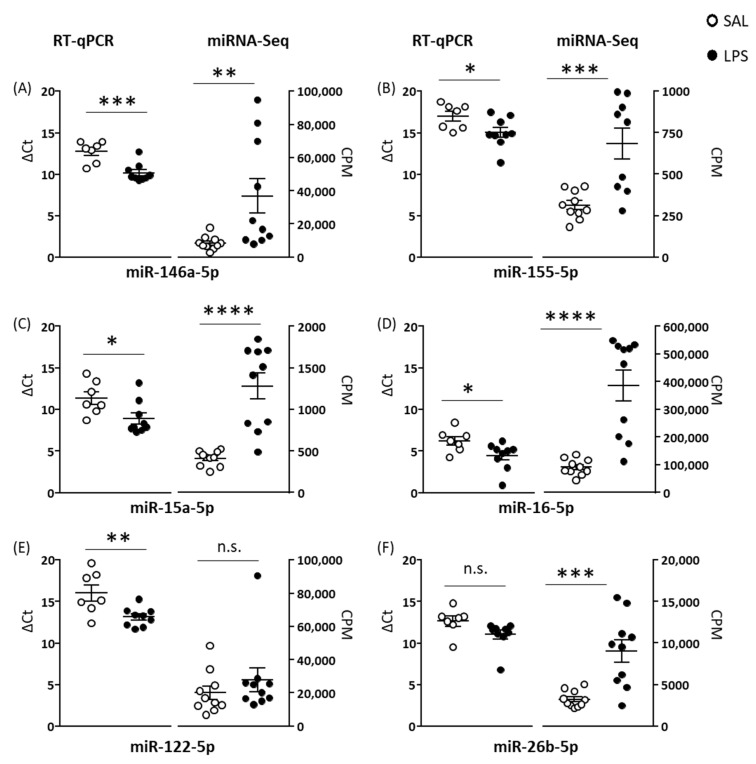
Comparison of the effects of lipopolysaccharide (LPS) on EV miRNA expression as determined by using RT-qPCR or miRNA-Seq. In two separate experiments conducted on two different cohorts, mice underwent LPS (1 mg/kg) or physiological saline (SAL) i.p. injection and after 5 h blood was collected and EVs isolated from plasma. In one experiment, 7 selected miRNAs were quantified using RT-qPCR, and in the case of 5 there was a significant increase in fold change. In the other experiment, miRNA-Seq was conducted. (**A**–**D**) Four of the five miRNAs upregulated in RT-qPCR were upregulated in miRNA-Seq: (**A**) miR-145a-5p, (**B**) miR-155-5p, (**C**) miR-15a-5p, and (**D**) miR-16-5p. (**E**) One of five miRNAs upregulated in RT-qPCR was not upregulated in miRNA-Seq: miR-122-5p. (**F**) One miRNA not upregulated in RT-qPCR was upregulated in miRNA-Seq: miR-26b-5p. ΔCt: normalized cycle threshold. CPM: normalized counts per million. Individual values, mean, and standard error of the mean (S.E.M) values are given. * *p* < 0.05, ** *p* < 0.01, *** *p* < 0.001, **** *p* < 0.0001, unpaired two-tailed Student’s *t*-test.

In the main miRNA-Seq study, effects of LPS versus SAL on the plasma EV miRNome after 5 h were investigated with *n* = 10 per group. The plasma EV total RNA concentration was assessed and was higher in LPS mice (mean = 4044 pg/µL) than SAL mice (mean = 409 pg/µL) (t(18) = 3.06, *p* = 0.007; Figure S2A). Primary miRNA quantification identified 1001–1879 (mean = 1697) mature miRNAs per sample. Differential expression analysis (*p* < 0.01, log_2_ fold change ≥1 or ≤ −1) identified 124 plasma EV miRNAs, 122 upregulated and two downregulated in LPS mice (81 upregulated miRNAs after false discovery rate (FDR) correction: adjusted *p* = 1 × 10^−16^–0.05) (Table S1). Four of the five miRNAs identified as upregulated by LPS using RT-qPCR in the pilot study samples were also upregulated by LPS using miRNA-Seq in the main study samples (Figure 1). Target-prediction and pathway analyses of differentially expressed miRNAs in the plasma EV miRNome were conducted. For the 15/122 most upregulated miRNAs in LPS mice, 1583 predicted target transcripts were identified, and 387 of these contributed to 70 significantly enriched KEGG pathways (Figure S3A). Regarding pathway functional annotation, signal transduction, nervous system, endocrine system, viral infection, cellular community, transport and catabolism, cell growth and death, and immune system pathways were identified. Interesting examples of target pathways include TNF and MAPK signalling (signal transduction); cholinergic, dopaminergic, and GABAergic synapse (nervous system); and T cell receptor signalling, Fc epsilon RI signalling, leukocyte trans-endothelial migration, and Th1-Th2 cell differentiation (immune system).

For the CSS versus CON experiment, the effects of 15-day CSS versus control handling (CON) on plasma EV miRNome at day 16 were investigated with *n* = 14 per group. The plasma EV total RNA concentration was assessed and was similar in CSS (mean = 368 pg/µL) and CON mice (mean = 361 pg/µL) (t(26) = 1.02, *p*= 0.30; Figure S2B). Per sample, 1196–1873 (mean = 1671) mature miRNAs were identified. Differential expression analysis identified 26 plasma EV miRNAs, with six upregulated and 20 downregulated in CSS mice (one upregulated and six downregulated miRNAs after FDR correction: adjusted *p* = < 1 × 10^−16^–0.05) (Table S3). Of the 20 EV miRNAs that were downregulated in CSS compared with CON mice, 10 belonged to the 122 that were upregulated in LPS versus SAL mice (Figure S4). For the six upregulated EV miRNAs in CSS mice, 497 predicted targets were identified and of these 50 contributed to 11 significantly enriched pathways (Figure S3B). For the 15/20 most downregulated miRNAs, 1100 predicted targets were retrieved, 112 of which contributed to 10 significantly enriched pathways (Figure S3C). With respect to pathway functional annotation, nervous system and metabolic disease were identified for the predicted targets of both upregulated and downregulated miRNAs in CSS mice, whilst the endocrine system was specific to targets of upregulated miRNAs, and signal transduction and cellular community were specific to targets of downregulated miRNAs. Lastly, separate target mining and pathway analysis were conducted for the 10 miRNAs that were both upregulated in LPS mice and downregulated in CSS mice (Figure S3D): 29 significantly enriched pathways were retrieved, including eight signal transduction pathways (MAPK, Ras, cGMP-PKG, mTor, Hedgehog, Hippo, JAK-STAT, and FoxO signalling pathways).

### 2.2. Experiment 2: Peripherally Administered EVs Exert Mild Effects on Body Weight, Immune Status, and Reward-Directed Behaviour

**Figure 2:**
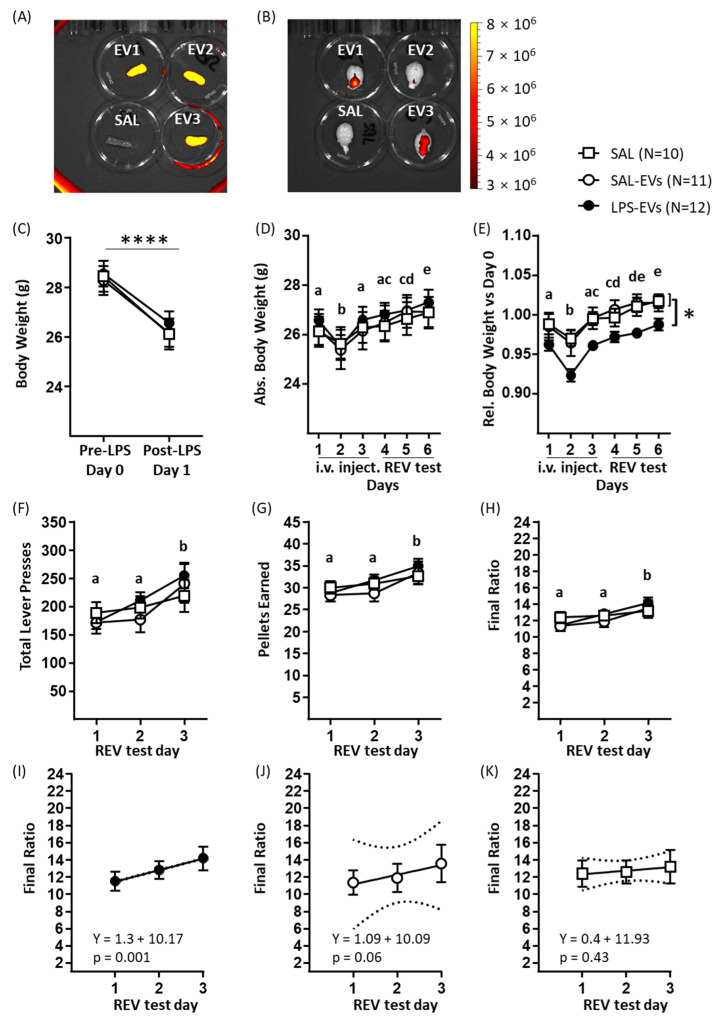
Effects of repeated peripheral injection of plasma/lymph node EVs on physical status and reward motivation in recipient mice. (**A**,**B**) Pilot experiment to assess biodistribution of EVs injected via the tail vein. EVs were labelled with ExoGlow dye and injected into recipient mice, and control recipient mice were injected with physiological saline only. Representative images obtained with the IVIS Lumina XR System of (**A**) spleens and (**B**) brains collected from 3 mice injected with ExoGlow-labelled EVs (EV1, EV2, EV3) and 1 control mouse (SAL). Signal intensity scale depicts radiant efficiency ((p/sec/cm^2^/sr)/(µW/cm^2^)). (**C**–**K**) Data for main study recipient mice. Data are mean ± S.E.M. unless otherwise stated. (**C**) Body weight prior to and 16 h after LPS injection (0.5 mg/kg, i.p.). Two-way RM ANOVA (Group × Day) yielded a significant day main effect. **** *p* < 0.0001. Mice are assigned to their groups although at these two timepoints they had not yet been injected with EVs or saline. (**D**) Absolute body weight during 3 days of injection and 3 days of behavioural testing. Two-way RM ANOVA (Group × Day) yielded no effect of group and a significant day main effect and post hoc Tukey’s test identified that body weight was lowest on day 2 and then increased consistently; days significantly different from each other are denoted by different letters (*p* ≤ 0.05). (**E**) Relative body weight on days of injection and of behavioural testing as a proportion of body weight at day 0. Two-way RM ANOVA (Group × Day) yielded a significant group main effect and Fisher’s LSD post hoc test identified that relative body weight was reduced in LPS-EV mice versus SAL-EV and SAL mice, which did not differ. There was also a significant day main effect, similar to that observed for absolute body weight. * *p* < 0.05. (**F**–**J**) Behavioural measures in the 3 reward-to-effort valuation (REV) tests using a progressive ratio schedule: (**F**) Total lever press responses. (**G**) Number of pellets earned. (**H**) Final ratio attained. For each measure, two-way RM ANOVA (Group × Test day) yielded a significant test day main effect and post hoc Tukey’s test identified that measures of reward-to-effort valuation were higher in test 3 than tests 1 and 2, as denoted by different letters (*p* ≤ 0.05). (**I**,**J**) Linear regression analysis for final ratio attained against test day in the REV test: (**I**) LPS-EV mice, (**J**) SAL-EV mice, and (**K**) SAL mice. Data are expressed as mean ± 95% confidence interval, the solid line is for the regression equation and the dotted curves indicates ± 95% confidence interval of the regression equation. The regression was significant for LPS-EV mice specifically.

Firstly, a pilot study was conducted to investigate the biodistribution of EVs injected intravenously in mice that had received low-dose LPS to increase BBB permeability. Following labelling of isolated plasma EVs with a fluorescent dye (ExoGlowTM-Vivo EV Labeling Kit, Near IR; System Biosciences, Palo Alto, CA, USA) and their tail vein injection in recipient mice, in vivo imaging demonstrated that signal intensity was highest in the injection site, upper abdomen, and head (Figure S5A–D). Given that high signal intensity in the tail could indicate a partial extra-venous injection [38], an experimenter with extensive experience in mouse tail vein injections conducted i.v. injections in Experiment 2. Subsequent ex vivo imaging of dissected organs indicated high EV-specific signal in spleen (Figure 2A) and liver (data not shown) and low EV-specific signal in brain (Figure 2B). Within the brain, in the case of EV1 in Figure 2B, plasma EVs were concentrated caudally, suggesting that mechanisms additional to diffusion across the BBB contribute to EV distribution.

**Figure 3:**
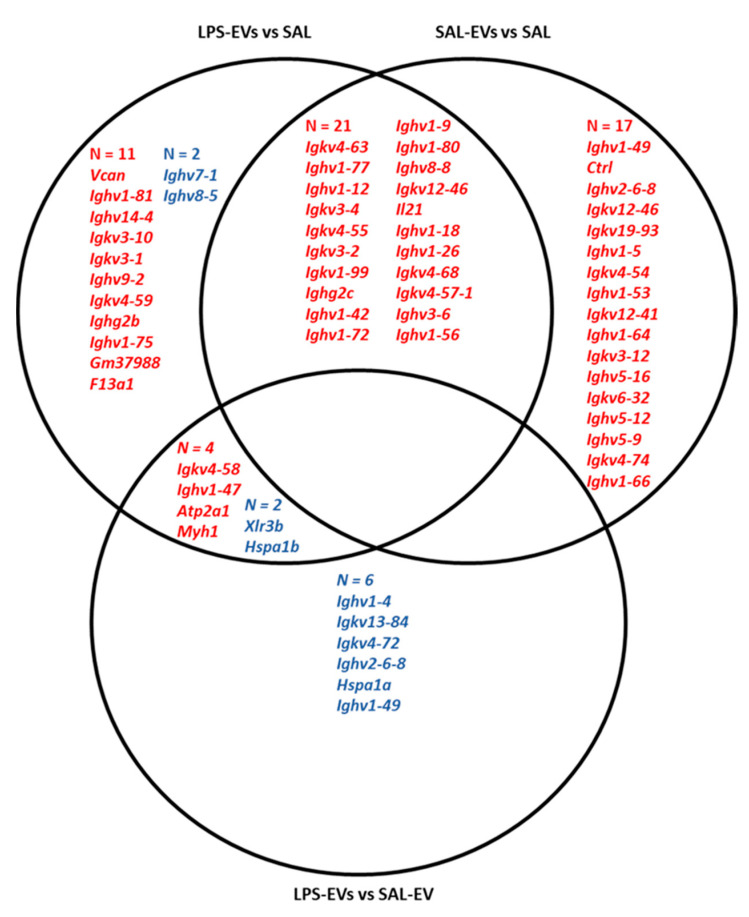
Venn diagram depicting the spleen tissue mRNAs identified as differentially expressed in pair-wise comparisons LPS-EVs versus SAL, SAL-EVs versus SAL, and LPS-EVs versus SAL-EVs (*p* < 0.01, log_2_ fold change ≥1 or ≤−1). Transcripts indicated in red were upregulated and those in blue were downregulated in the comparison indicated. Therefore, 21 transcripts were upregulated in both EV-infused groups compared with SAL-infused mice, and 4 transcripts were upregulated and 2 downregulated in LPS-EV mice compared with SAL-EV- and SAL-infused mice.

For the main study, recipient mice were administered low-dose LPS on the day prior to the first daily injection; then, on three consecutive days, they were injected i.v. with saline only (SAL) or with isolated plasma/lymph node EVs obtained from donor mice that had been injected with either LPS (1 mg/kg, LPS-EVs) or saline (SAL-EVs). Throughout this period and subsequent behavioural testing, mice were given 70–150% of their baseline food consumption per day with the aim of maintaining their body weight at 95–100% of baseline. Mice in the three groups received a similar amount of daily food (overall mean food given per day: SAL 3.69 ± 0.34 g, SAL-EVs 3.86 ± 0.33 g, LPS-EVs 3.95 ± 0.27 g: F(2, 31) = 0.58; *p* = 0.57). Using body weight as a marker for physical state, this was decreased on the day after LPS administration, as measured immediately prior to the first EV/SAL injection (day main effect: (F(1, 31) = 627.6, *p* < 0.0001; Figure 2C), and with no effect of prospective EV group allocation (*p* = 0.50). Body weight was then measured on each of the three injection days (1–3) and the subsequent three days of behavioural testing (4–6) prior to injection or testing. In terms of absolute body weight, this decreased on day 2 relative to day 1 and recovered progressively to pre-injection levels over the following days (day main effect: (F(5, 150) = 45.5, *p* < 0.0001, followed by post hoc Tukey’s test; Figure 2D); there was no effect of group, neither main effect nor in interaction with day (*p* ≥ 0.50). In terms of body weight expressed relative to baseline body weight (prior to LPS administration, day 0), this was lower in LPS-EV mice than SAL-EV mice and SAL mice (group main effect: F(2, 30) = 3.82, *p* = 0.03, followed by post hoc Fisher’s LSD test: LPS-EVs versus SAL-EVs: *p* = 0.022; LPS-EVs versus SAL: *p* = 0.028; Figure 2E). As for absolute body weight, in all groups, relative body weight decreased on day 2 and then recovered (day main effect: (F(5, 150) = 50.76, *p* < 0.0001, followed by post hoc Tukey’s tests; Figure 2E). After completion of 3 days of injection, mice underwent three daily tests of sucrose reward motivation on a progressive ratio schedule. In all groups, the measures of sucrose reward motivation were increased in test 3 compared to tests 1 and 2, which included the following: number of lever presses (test main effect: F(2, 60) = 11.74, *p* < 0.0001, post hoc Tukey’s test; Figure 2F), number of pellets earned (test main effect: F(2, 60) = 13.12, *p* < 0.0001, post hoc Tukey’s test; Figure 2G), and final ratio attained (test main effect: F(2, 60) = 11.73, *p* < 0.0001, post hoc Tukey’s test; Figure 2H). For group, there was neither a main nor interaction effect for these behavioural measures (*p* ≥ 0.30). Using a posteriori linear regression of behaviour against test day, it was nonetheless the case that behaviour was predicted by test day in the case of LPS-EV mice but not of SAL-EV or SAL mice. For example, for final ratio attained, the regression was significant for LPS-EV mice (r = 0.53; F(1, 34) = 13.91, *p* = 0.001; Figure 3I) and not for SAL-EV mice (r = 0.33; F(1, 31) = 3.82, *p* = 0.06; Figure 2J) or SAL mice (r = 0.15; F(1, 28) = 0.65, *p* = 0.43; Figure 2K). This was also the case for total lever presses (Figure S6A–C) and pellets earned (Figure S6D–F). This indicates that the effect of test day on the increase in sucrose reward motivation was more robust in LPS-EV mice than SAL-EV and SAL mice.

One day after completion of behavioural testing and, therefore, four days after the last injection, blood and spleen samples were collected. In blood plasma, the concentrations of 19 cytokines or chemokines were determined using electrochemiluminescence immunoassay. Plasma concentrations of 13 cytokines/chemokines were on the corresponding standard curve but in no case was there a significant difference between groups (one-way ANOVAs, *p* = 0.08–0.81). Spleen tissue was processed for mRNA-Seq and differential expression analysis was conducted with significance threshold *p* < 0.01 and log_2_ fold change ≥1 or ≤−1 (no gene was differentially expressed after FDR correction; adjusted *p* ≥ 0.1) (Table S3). As provided in Figure 3, for the comparison LPS-EVs versus SAL-EVs, 12 transcripts were differentially expressed and four upregulated and eight downregulated in LPS-EV mice spleens. For LPS-EV versus SAL recipient mice, 40 transcripts were differentially expressed and 36 upregulated and four downregulated in LPS-EV mice spleens. The four upregulated transcripts in LPS-EV versus SAL-EV mice were also upregulated in LPS-EV versus SAL mice. For SAL-EV versus SAL recipient mice spleens, 38 transcripts were upregulated in SAL-EVs. There were 21 transcripts upregulated in both LPS-EV and SAL-EV compared with SAL: these genes encode for heavy and light chain variable domains of immunoglobulins involved in phagocytotic and immune response processes, especially B cell activation and B-cell receptor signalling.

### 2.3. Experiment 3: Nucleus Accumbens-Infused LPS-Activated EVs Exert a Mild Effect on Reward-Directed Behaviour and Increase Expression of NAc Immune-Inflammatory Pathway Transcripts

**Figure 4:**
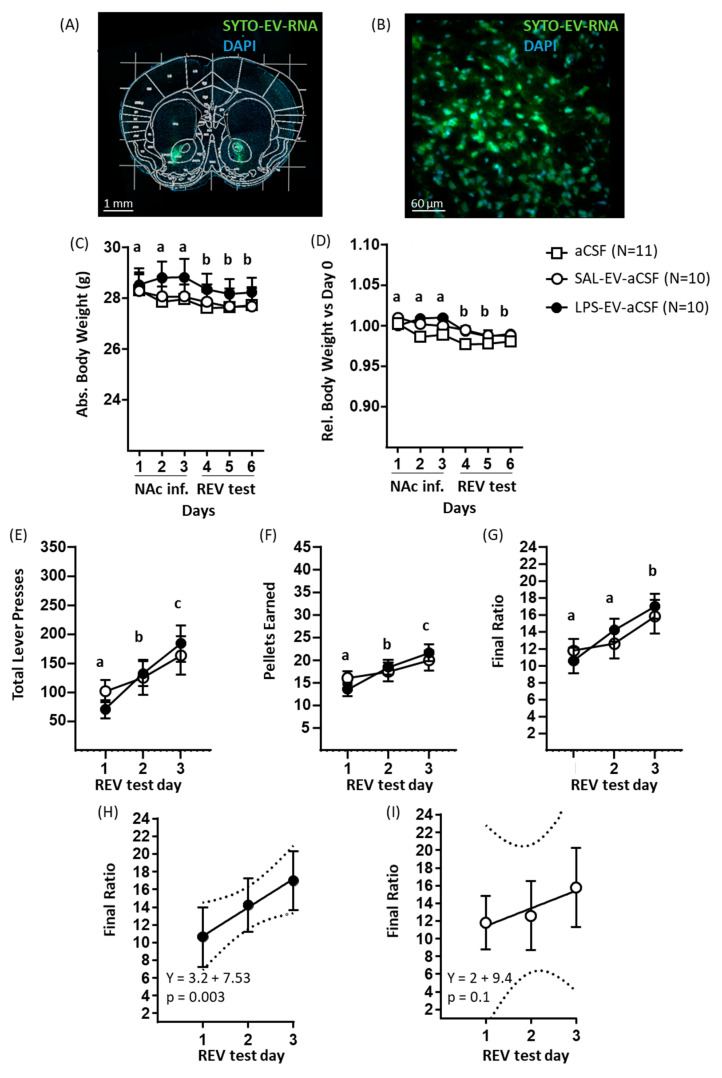
Effects of repeated infusion of plasma EVs onto nucleus accumbens on physical status and reward motivation in recipient mice. (**A**,**B**) Histological assessment of the site of infusion of EVs with SYTO dye-labelled RNA onto NAc. (**A**) Representative epifluorescence microscope image of coronal brain section at +1.18–0.98 mm relative to bregma showing SYTO-EV RNA (green) and DAPI-labelled cell nuclei (blue). Magnification 5×. (**B**) The same brain section at 40× magnification at the infusion site showing co-localization of SYTO-labelled EV RNA (green) and NAc cell nuclei (blue). (**C**–**I**) Data for main study recipient mice. Data are mean ± S.E.M. unless otherwise stated. (**C**) Body weight during 3 days of infusion and 3 days of behavioural testing. Two-way RM ANOVA (Group × Day) yielded no effect of group and a significant day main effect; post hoc Tukey’s test identified that body weight was lower on test days than on infusion days, denoted by different letters (*p* ≤ 0.05). (**D**) Body weight on days of infusion and of behavioural testing as a proportion of body weight at day 0. Two-way RM ANOVA (Group × Day) yielded no effect of group and a significant day main effect, with a decrease during testing relative to infusion days as for absolute body weight. (**E**–**I**) Behavioural measures in the 3 reward-to-effort valuation (REV) tests using a progressive ratio schedule conducted with LPS-EV-aCSF and SAL-EV-aCSF recipient mice: (**E**) Total lever press responses. (**F**) Number of pellets earned. (**G**) Final ratio attained. For each measure, two-way RM ANOVA (Group × Test day) yielded a significant test day main effect and post hoc Tukey’s test identified an overall increase in measures of reward-to-effort valuation from test to test, as denoted by different letters (*p* ≤ 0.05). (**H**,**I**) Linear regression analysis for final ratio attained against test day in the REV test: (**H**) LPS-EV-aCSF mice and (**I**) SAL-EV-aCSF mice. Data are expressed as mean ± 95% confidence, the solid line is for the regression equation, and the dotted curves indicate ± 95% confidence interval of the regression equation. The regression was significant for LPS-EV mice specifically.

Firstly, a pilot study was conducted to investigate whether EV RNA infused into the NAc was taken up into cells and, if so, into which cell types. EVs isolated from plasma were labelled with a fluorescent dye specific for RNA and infused into the NAc on 3 consecutive days. In coronal sections, it was apparent that the EV RNA fluorescent signal was present in and restricted to cell bodies in the NAc tissue at the infusion site (Figure 4A,B). Immunofluorescence staining for cell type-specific protein markers revealed extensive co-localization of EV RNA fluorescence with the neuronal marker NeuN (Figure S7A) and the mature oligodendrocyte marker Olig2 (Figure S7B), whilst co-localization was relatively sparse with the microglia marker CD68 (Figure S7C) and the astrocyte marker GFAP (S7D).

On the basis of the validatory evidence that EVs, or at least their RNA cargo, enter brain cells, the main study of the effects of NAc EV infusion was conducted. NAc-cannulated recipient mice were administered 3 daily infusions of aCSF only, or of plasma EVs isolated from donor mice injected with LPS (1 mg/kg, LPS-EV-aCSF) or of SAL (SAL-EV-aCSF). Throughout this period, mice were given 70–150% of their baseline food consumption per day, with mice in the three groups receiving a similar amount of daily food (overall mean food given per day: aCSF 3.94 ± 0.17 g, SAL-EV-aCSF 3.98 ± 0.15 g, LPS-EV-aCSF 4.32 ± 0.27 g: F (2, 29) = 2.04; *p* = 0.15). The body weight of recipient mice was measured on each of the three infusion days (1–3) and the subsequent three days of behavioural testing (4–6), prior to infusion or testing. Absolute body weight (Figure 4C) was lower on test days relative to infusion days (day main effect: F(5, 140) = 13, *p* < 0.0001, post hoc Tukey’s test), and in the absence of a main or interaction effect of group (*p* ≥ 0.10). For body weight expressed relative to baseline on day 0 (Figure 4D), the findings were similar, with lower values on test days relative to infusion days (day main effect: (5, 140) = 12.21, *p* < 0.0001, post hoc Tukey’s test) in the absence of group differences (*p* ≥ 0.09). In the three daily tests of sucrose reward motivation on a progressive ratio schedule conducted with the two groups that received EVs in the NAc, there was an overall day-by-day increase in the measures of sucrose reward motivation, which included the following: number of lever presses (test main effect: F(2, 36) = 13.02, *p* < 0.0001, post hoc Tukey’s test; Figure 4E); number of pellets earned (test main effect: F(2, 36) = 12.94, *p* < 0.0001, post hoc Tukey’s test; Figure 4F); and final ratio attained (test main effect: F(2, 36) = 13.03, *p* < 0.0001, post hoc Tukey’s test; Figure 4G). There was no group main or interaction effect for these motivational measures (*p* ≥ 0.80). Using a posteriori linear regression of behaviour against test day, it was nonetheless the case that behaviour was predicted by test day in the case of LPS-EV-aCSF mice but not of SAL-EV-aCSF mice. For example, for final ratio attained, the regression was significant for LPS-EV-aCSF mice (r = 0.52, F(1, 28) = 10.37, *p* = 0.003; Figure 4H) and not for SAL-EV-aCSF mice (r = 0.30, F(1, 28) = 2.82, *p* = 0.10; Figure 4I). This was also the case for total lever presses (Figure S8A,B) and pellets earned (Figure S8C,D). This indicates that the effect of test day on increasing sucrose reward motivation was more robust in mice that were infused in NAc with EVs derived from LPS-challenged mice.

**Figure 5:**
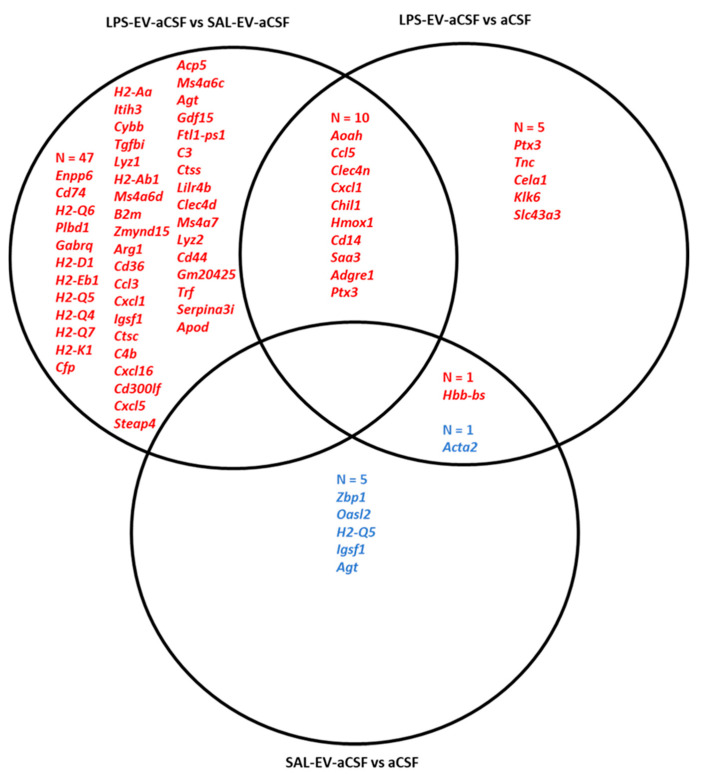
Venn diagram depicting the NAc tissue mRNAs identified as differentially expressed in pair-wise comparisons LPS-EV-aCSF versus SAL-EV-aCSF, LPS-EV-aCSF vs. aCSF, and SAL-EV-aCSF vs. aCSF (*p* < 0.01, log_2_ fold change ≥1 or ≤−1). Transcripts indicated in red were upregulated and those in blue were downregulated in the comparison indicated. Therefore, 10 transcripts were upregulated in LPS-EV-aCSF mice compared with both SAL-EV-aCSF and aCSF mice, whilst one transcript was upregulated and one was downregulated in both EV-infused groups compared with aCSF mice.

One day after completion of behavioural testing and/or 4 days after the last NAc infusion, brains from mice in all three groups were collected for microdissection of NAc tissue and processing for RNA-Seq. As a positive validation of the methodology, in line with NAc neurons being primarily GABA medium spiny neurons, the expression of the GABA neuron-specific marker gene Gad1 was considerably higher (mean counts per million: 13,962) than that of the glutamate neuron-specific marker Slc17a7 (mean counts per million: 306) [39]. The genes for nucleus accumbens-associated protein 2 (Nacc2) and dopamine receptors 1 and 2 (Drd1, Drd2) were also highly expressed, in line with expectations [40,41]. In differential expression analysis of the NAc transcriptome (Table S4), conducted with significance threshold *p* < 0.01 and log_2_ fold change ≥1 or ≤−1, in LPS-EV-aCSF versus SAL-EV-aCSF mice, 57 genes were identified, all of which were upregulated in NAc from LPS-EV-aCSF mice (three of these genes were also differentially expressed after FDR correction; adjusted *p* = 0.0006–0.04). In comparison LPS-EV-aCSF versus aCSF, 16 genes were identified and 15 upregulated and one downregulated in NAc from LPS-EV-aCSF mice (FDR adjusted *p* ≥ 0.2). In SAL-EV-aCSF versus aCSF mice, seven genes were identified and one upregulated and six downregulated (FDR adjusted *p* ≥ 0.7). As given in Figure 5, 10 NAc genes were upregulated in LPS-EV-aCSF mice compared with both SAL-EV-aCSF and aCSF mice. Over-representation pathway analysis conducted with these 10 genes identified 33 significant Gene Ontology-Biological Processes pathways, including pathways associated with *inflammatory response*, *defence response*, *leukocyte activation, migration, and leukocyte-mediated immunity*; *cytokine production*; and *LPS-mediated signalling* (Table 1). Based on miRWalk, none of these 10 genes was identified as being a potential target of the miRNAs identified as upregulated in LPS-challenged EVs in Experiment 1.

## 3. Discussion

This mouse study was conducted to investigate the iterative hypotheses that acute-high and chronic-low inflammation induce changes in the plasma EV miRNome in mice and that inflammation-activated peripheral EVs from donor mice affect physical status, peripheral and CNS inflammatory status, and gustatory reward processing of recipient mice upon peripheral or central administration. In order to explore these hypotheses, we conducted a first experiment with the aims to investigate and compare the effects of acute, systemic LPS and 15-day CSS on the plasma EV miRNome (Experiment 1). Then, having obtained evidence that LPS induces substantial changes in the plasma EV miRNome, we conducted two donor-recipient transfer experiments with the aims to investigate and compare the effects of peripheral EVs collected from donor mice treated with LPS or SAL and delivered to the periphery (Experiment 2) or brain (Experiment 3) of recipient mice.

In Experiment 1, total RNA concentration was higher in plasma EVs isolated from LPS than SAL mice, suggesting either increased RNA/miRNA loading within EVs or increased EV release from cells. LPS induced upregulation of 122 EV miRNAs and downregulation of two EV miRNAs. LPS-induced upregulation of specific EV miRNAs has been reported in vitro [42] and in vivo [27]. Li et al. reported six of seven serum EV miRNAs that they measured were upregulated at 6 h after LPS, and four of these—miR-15a, miR-15b, miR-146a, and miR-155—were upregulated in this study; each of these miRNAs is involved in inflammatory processes [43,44]. In addition, several more of the plasma EV miRNAs upregulated in LPS mice in this study are involved in the innate immune response mediated by toll-like receptor 4 at which LPS is an agonist, including let-7a, let-7i, miR-19, miR-221, and miR-222 [15]. Turning to the CSS experiment, six miRNAs were upregulated and 20 were downregulated in CSS compared to CON mice. This contrast between LPS and CSS effects is further emphasized by the finding that 10 of the 20 EV miRNAs downregulated by CSS were upregulated by LPS. The target transcripts of these miRNAs were enriched for several signal transduction pathways. This suggests the involvement of these miRNAs in both acute-high and chronic-low inflammatory states, which are upregulated and possibly contributing to the activation of immune-inflammatory signalling pathways during acute inflammation and downregulated and possibly contributing to a negative feedback loop during chronic inflammation. Indeed, most miRNAs can have both pro-inflammatory and anti-inflammatory functions [15], and the overall effect of miRNA-mediated regulation depends also on the type and activation status of the target cell [45]. The rationale for including the CSS manipulation in this study was that it results in changes in aversion and reward processing relevant to those that occur in the stress-related neuropsychiatric disorder MDD and also activates pro-inflammatory processes [25,26,33,34,46]. In this regard, it is noteworthy that, of the modest total number of EV miRNAs dysregulated by CSS, several miRNAs that were either upregulated—miR-34c and miR-184—or downregulated—let-7a, -7b, -7f, miR-26a/b-5p, miR-103-5p, miR-190a-5p, and miR-374b-5p—in CSS mice have been reported to be dysregulated (upregulated or downregulated) in blood, serum, plasma, peripheral blood mononuclear cells [47,48], or serum EVs [22] of MDD patients compared to control subjects. Our findings on the effects of LPS and CSS suggest that the EV miRNome will be more dysregulated in disorders associated with acute peripheral and CNS inflammation, including autoimmune CNS disorders, than in stress-related neuropsychiatric disorders. To the best of our knowledge, nine studies have been published so far on the peripheral EV miRNome of MS patients [49,50], and two in MDD patients, of which one is a case study [22,51]. Due to the limited number of studies and high heterogeneity of methods, a comparison of the changes in the peripheral EV miRNome between the two diseases is currently not informative. However, recent studies conducted by our group on the serum EV miRNome of MS and MDD patients compared with their respective controls and using the same experimental protocol as that applied here indicate marked and moderate changes in MS and MDD, respectively (unplublished data), which is consistent with our findings in mice.

Given the strong upregulation of plasma EV miRNAs by LPS, this method of immune activation was selected for subsequent donor–recipient mouse experiments. Given the short half-life of LPS [52] and the previously reported absence of LPS in serum EVs collected at 1–24 h after systemic LPS administration [27], it is unlikely that EVs administered to LPS-EV mice contained LPS. The overall aim of these experiments was to add to the small and interesting current body of evidence for the effects of increased EV load on physiology and behaviour. This included comparison of exogenous LPS-activated EVs versus baseline-state EVs versus no EVs, and comparison of exogenous EVs administered to the periphery (Experiment 2) or the brain (Experiment 3). Based on our quantification of plasma EV RNA, LPS-EV recipient mice received a higher amount of EV RNA/miRNA cargo and possibly also a higher number of EVs than SAL-EV recipient mice. Another aspect of the study design that is important to consider is that recipient mice of Experiment 2 received a low dose of LPS prior to EV injection in the attempt to increase EV BBB passage in both directions. With respect to the effects on physical status, peripheral injection of LPS-EVs resulted in a small decrease in relative body weight compared to the injection of SAL-EVs or SAL. Loss of body weight is one of the symptoms of LPS-induced sickness status [53]. Therefore, our findings suggest that EVs might contribute to the mediation of this effect of LPS. To the best of our knowledge, this is the first report of the study of effects of exogenous immune-activated EVs on body weight. In contrast, central infusion of LPS-EV-aCSF did not alter body weight compared to SAL-EV-aCSF or aCSF only. This might be due to the effects of targeted EV infusion, i.e., onto the NAc, being restricted to the local environment, whilst peripherally injected EVs are taken up into different organs and, therefore, would be expected to exert more widespread effects.

With regards to EV effects on peripheral inflammatory status, as investigated in mice that received i.v. injection of SAL only or of EVs isolated from donor mice challenged with LPS (1 mg/kg) or SAL, there was no difference between groups in the plasma protein levels of several pro-inflammatory cytokines (including IL-6 and TNF-α) and chemokines. In contrast, previous studies have reported changes in blood inflammatory markers upon peripheral infusion of immune-activated EVs from donor mice. These include increased blood levels of IL-6 and TNF-α mRNA when donor mice received 0.5 or 10 mg/kg LPS, and increased serum levels of TNF-α protein when donor mice received 10 but not when they received 0.5 mg/kg LPS [27]. When mice received peripheral injection of EVs that were isolated from MDD patients and, therefore, potentially immune activated, they displayed increased serum levels of oxidative stress markers [22]. In both of these studies, ex vivo assessment was conducted 24 h after EV administration, whilst here recipient mice were sacrificed four days after the last EV infusion, suggesting that the effects of exogenous immune-challenged EVs on transcription/translation in the target cells might be transitory. Moreover, a somewhat higher amount of EVs was injected in these two studies compared to the present study: here, mice received three injections each of an estimated 4 × 10^10^ EVs; in Wei et al., mice received four injections of about 1.4 × 10^11^ EVs, and in Li et al. mice received a single injection of 1 mg EVs which approximates to 2 × 10^12^ particles [54] whilst a dose of 500 ng EVs did not induce a neuroinflammatory response. The spleen has been reported as a major site of accumulation of peripherally administered exogenous EVs [55,56]; therefore, we considered it appropriate to investigate this organ to assess EV effects. Whilst there were minimal differences between spleen transcriptomes of LPS-EV and SAL-EV mice, each of these two EV groups underwent upregulation of a number of immune-related genes, and indeed many of the same genes, compared with SAL mice. The spleen plays an important role in immune surveillance, including activation of immune-inflammatory pathways in response to damage-associated and pathogen-associated molecular patterns [57,58]. As reviewed by Buzas et al., extracellular miRNAs—including miRNAs released by EVs—can act agonistically at toll-like receptors on immune cells, thereby triggering immune activation [13]. Therefore, these findings might suggest that exogenous EVs, potentially including their miRNA cargo, can stimulate spleen macrophages triggering a prolonged immune response, which, based on the gene pathway analysis, might involve B cell activation and antibody production. With respect to central inflammatory status, LPS-EV-aCSF infused into the NAc significantly affected the latter’s transcriptome, increasing the mRNA levels of several genes compared to mice injected in the NAc with SAL-EV-aCSF or aCSF. These findings provide further evidence of uptake of EVs and/or their functionally active molecular cargo into NAc cells, as demonstrated here by histological assessment of NAc tissue from mice infused with RNA-labelled plasma EVs. Transcriptional changes induced by uptake of functionally active EV cargo upon central infusion have been reported [8]. Interestingly, a more marked effect was observed between LPS-EV-aCSF mice compared with SAL-EV-aCSF mice than with aCSF mice in terms of the number of differentially expressed genes: This was mainly due to upregulation of transcript expression in LPS-EV-aCSF mice compared with aCSF mice and downregulation of expression of the same transcripts in SAL-EV-aCSF mice compared with aCSF mice. Opposite effects of immune-challenged versus naïve EVs from the same cellular source have been reported [22,29,59], suggesting that these might be mediated by different/opposite transcriptional regulation. The 10 genes that were upregulated in LPS-EV-aCSF versus both SAL-EV-aCSF and aCSF mice were significantly enriched for immune-inflammatory pathways, including LPS-mediated signalling pathway, suggesting EV involvement in such processes. Brain (intracerebroventricular) infusion of LPS-activated EVs induced pro-inflammatory status in microglia and astrocytes [27].

Acute, systemic LPS administration has been shown to decrease absolute sucrose intake [60,61,62] and sucrose versus water preference [53,63,64,65]. In order to obtain data on the motivation for sweet-tasting reward under effortful conditions, operant reinforcement protocols can be introduced. Using this approach with a high cost, high value reward/low cost, low value reward choice-schedule—chocolate-sucrose pellets on fixed-ratio 10/grain diet on fixed ratio 1—it was demonstrated that systemic LPS results in reduced absolute effort for reward but to a relative increase in effort for high cost, high value reward at 24 h [32]. These findings suggest that LPS induces sickness-related increased reward-to-effort valuation but does not affect preference for sweet reward [32,66]. At 48 h post-LPS injection, test performance had normalized; this temporal limitation of LPS effects on reward-directed behaviour is consistent with that of other non-effortful tests, e.g., sucrose preference, with LPS effects persisting for between 24 h and 48 h [67]. Here, using a progressive ratio (PR) schedule of reinforcement for chocolate-sucrose pellets and starting the behavioural testing at 1 day after the last EV/vehicle administration, we show that neither peripheral nor central administration of LPS-challenged EVs reduced sucrose reward-to-effort valuation compared to matched control groups. This suggests that EVs do not directly contribute to the mediation of the effects of LPS on reward-to-effort valuation. Quite the contrary, in both systemic and NAc experiments, all groups showed a test day-by-test day increase in reward-to-effort valuation; as they had undergone only one prior test with PR schedule prior to testing EV effects, this finding could reflect improved learning of the response–reward association as well as increased reward-to-effort valuation across tests. In addition, for each test measure, the amount of increase was predicted by test day for LPS-EV mice but not for SAL and SAL-EV mice and for LPS-EV-aCSF mice but not for SAL-EV-aCSF mice. These findings might reflect that a prolonged, mild state of sickness is induced by LPS-activated EVs administered to the periphery—relative body weight was reduced in LPS-EV mice—or to the NAc, and that this is followed by recovery from this state and a return to homeostasis that requires high energy intake, even under effortful conditions. For the EV-NAc behaviour experiment, the absence of a non-EV control limits the conclusions that can be drawn. Nonetheless, the predictive association between test day and reward-to-effort valuation in LPS-EV-aCSF mice specifically co-occurred with an upregulation of genes in the NAc transcriptome relative to SAL-EV-aCSF mice. Upregulation of ten of these genes was replicated relative to aCSF mice, and these genes were involved in immune and inflammatory response pathways, including the LPS-mediated signalling pathway. Comparing the current behavioural findings with those of previous studies of EV administration, peripheral injection of IFN-β-activated serum EVs in naïve recipient rats elicited mild sickness behaviour in the form of decreased locomotion as measured soon after administration [30], and the peripheral infusion of serum EVs isolated from MDD patients in naïve recipient mice induced decreased activity in the forced swim and tail suspension tests, and increased novelty suppressed feeding as measured soon after administration [22].

This study’s findings have to be interpreted in light of several limitations. Firstly, in common with other EV studies, evidence for the tissue/cellular origin of the isolated plasma EVs is lacking. In Experiments 2 and 3, ex vivo analyses were conducted four days after the last EV administration and, therefore, possibly after the duration of some (LPS-)EV-mediated effects. Moreover, the experimental design of measuring several parameters in the same subjects necessitated that ex vivo analyses did not reflect the peripheral and central inflammatory status of recipient mice at the times of EV administration and behavioural testing. In this respect, assessment of the immune-inflammatory status of recipient mice during EV administration would provide relevant additional information. In Experiment 3, behavioural testing for reward motivation was not conducted with the non-EV control group, i.e., mice receiving aCSF-only in the NAc. Observed effects of EVs on the spleen and NAc transcriptomes were not validated by using other methods, e.g., PCR. Investigation of potential effects of peripheral EVs on central processes and vice versa is lacking. Lastly, it would be relevant to conduct functional assays to investigate whether specific EV miRNAs upregulated by LPS can recapitulate some of the transcriptional changes induced in the spleen or NAc by LPS-activated EVs.

In summary, our results demonstrate that whereas both LPS and CSS induce changes in the mouse plasma EV miRNome, the effect of the former is more marked and this despite the relative chronicity of the latter. Overall, donor–recipient EV transfer studies demonstrate that repeated exposure to exogenous EVs and particularly those carrying an LPS-activated cargo exerts effects on the physical, peripheral and central transcriptome and behavioural status of mice. The current data suggest the involvement of EVs in the pathophysiological pathways of inflammation-associated brain disorders.

## 4. Materials and Methods

### 4.1. Animals and Housing

All experiments except for the chronic social stress (CSS)—plasma EV miRNome experiment were conducted with adult male C57BL/6JRj mice supplied by Janvier (Le Genest-Saint-Isle, France); the CSS experiment was also conducted with adult male C57BL/6J mice, but they were bred in-house. Janvier mice arrived at age 7 weeks and all mice were aged 11–13 weeks at study onset. Mice were kept in littermate pairs throughout the experiment. For the CSS experiment, male ex-breeder CD-1 mice (Janvier) aged 32 weeks and caged singly were used. Mice were maintained on a reversed 12:12 h light-dark cycle (light off: 07:00–19:00) in an individually-ventilated caging system at 21–22 °C and 50–60% humidity. Complete-pellet diet (Provimi, Kliba Ltd., Wisconsin, IA, USA) and water were available ad libitum unless stated otherwise. All procedures were conducted during the dark phase of the cycle. The study was conducted under a permit for animal experimentation (ZH130/2017) issued by the Veterinary Office of Canton Zurich in accordance with the regulations of the Swiss Federal Veterinary Office. All efforts were made to minimize the number of mice studied and any unnecessary stress.

### 4.2. Experimental Designs

**Figure 6:**
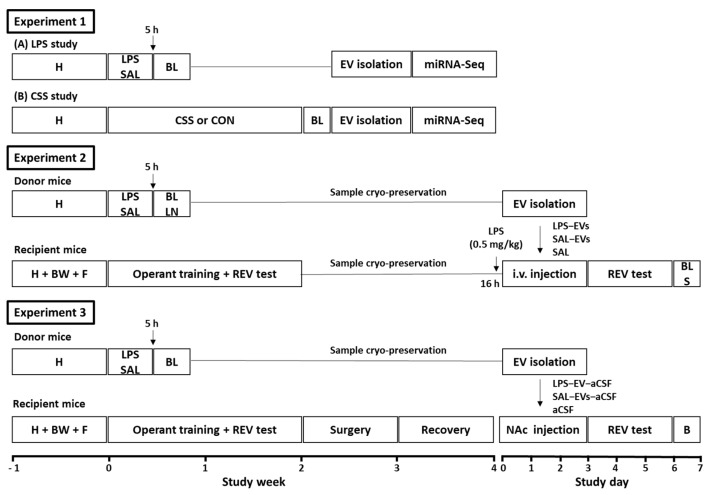
Designs of the three main and inter-related experiments of this study, each conducted with a different cohort of mice. Experiment 1. (**A**) Mice received a single i.p. injection of lipopolysaccharide (LPS, 1 mg/kg) or physiological saline (SAL), and blood (BL) was collected 5 h post-injection. (**B**) Mice were exposed to 15-day chronic social stress (CSS) or control handling (CON), and blood was collected 24 h later. In (**A**,**B**), EVs were isolated from plasma samples and microRNA-Sequencing was conducted. Experiment 2. Donor mice received a single i.p. injection of LPS (1 mg/kg) or SAL, and blood and lymph nodes (LN) were collected at 5 h post-injection for EV isolation. Recipient mice underwent daily handling (H) and determination of baseline body weight (BW) and food consumption (F) for 1 week; under food restriction, they were trained for 15 days for the operant test of reward-to-effort valuation (REV). At the end of training, mice underwent a REV test and were allocated to groups by counterbalancing on test scores. At 16 h prior to injections, recipient mice were injected with a low dose of LPS (0.5 mg/kg). Mice then received 3 daily intravenous injections of EVs isolated from the donor mice that received LPS (LPS-EVs) or SAL (SAL-EVs), or of physiological saline (SAL) only. On days 4–6 mice were tested in the REV test. On day 7, blood and spleen (S) were collected for measurement of pro-inflammatory biomarkers and RNA-Sequencing, respectively. Experiment 3. Donor mice received a single i.p. injection of LPS (1 mg/kg) or SAL and blood was collected 5 h post-injection for plasma EV isolation. Recipient mice were trained and tested for REV as in Experiment 2 and allocated to groups by counterbalancing test scores. Recipient mice were then fitted with a bilateral cannula onto the nucleus accumbens (NAc) followed by recovery for 7 days. Recipient mice received 3 daily infusions onto the NAc of EVs isolated from donor mice after LPS (LPS-EV-aCSF) or SAL (SAL-EV-aCSF) or of artificial cerebrospinal fluid (aCSF) only. On days 4–6, mice infused with LPS-EV-aCSF or SAL-EV-aCSF were tested in the REV test. On day 7, brains (B) were collected from all mice for NAc tissue RNA-Sequencing.

Three iterative experiments were conducted with adult male C57BL/6 mice, and experimental designs are summarised below and depicted in Figure 6. Experiment 1 investigated the effects of lipopolysaccharide (LPS) or chronic social stress (CSS) on the plasma EV miRNome. A first pilot study was conducted on the effects of acute systemic LPS on specific plasma EV miRNAs, which were selected based on their role in inflammation including LPS-activated toll-like receptor 4 signalling and in inflammation-associated disorders. Mice received a single intraperitoneal (i.p.) injection of LPS (1 mg/kg) or physiological saline (SAL), blood was collected after 5 h—the approximate peak time point of LPS-induced systemic inflammatory response—and plasma was purified for subsequent EV isolation. EVs were isolated using a precipitation-based method, non-EV RNA was removed, and RT-qPCR was conducted for the miRNAs of interest. In the main study, the effects on plasma EV miRNome of acute LPS at 5 h or of 15-day CSS at day 16 relative to their respective control groups (SAL, CON) were investigated using microRNA-Sequencing (miRNA-Seq). Plasma EVs were isolated using size exclusion chromatography in order to obtain purer EV preparations than with the precipitation-based method. Pair-wise differential expression analysis and target gene-pathway analysis of the plasma EV miRNomes were conducted for LPS versus SAL and for CSS versus CON.

Experiment 2 investigated the effects of peripheral, i.e., tail vein, injection of EVs isolated from plasma and lymph nodes of LPS-challenged donor mice on physical status, peripheral inflammatory markers, the spleen mRNome, and reward-directed behaviour, in recipient mice. A pilot study was conducted to determine the biodistribution of peripherally injected EVs. Plasma EVs from donor mice were isolated, fluorescently labelled, resuspended in physiological saline, and injected via the tail vein of recipient mice that had received low dose LPS (0.5 mg/kg) 16 h previously; in vivo and ex vivo imaging was then conducted. In the main experiment, donor mice (*n* = 40) received LPS (1 mg/kg; LPS-EVs) or SAL (SAL-EVs) and plasma/lymph nodes were collected after 5 h for EV isolation as in the pilot study. Recipient mice received low-dose LPS (0.5 mg/kg), followed on the next 3 days by i.v. injection of LPS-EVs (*n* = 12), SAL-EVs (*n* = 12) or physiological saline only (SAL, *n* = 10). For EV groups, each mouse was injected with EVs derived from the same pool and with an estimated 4 × 10^10^ EVs per day. Previous studies reported increased levels of inflammatory markers after 1 injection of 2 × 10^12^ or 4 injections of 1.4 × 10^11^ EVs [22,27]. On the next 3 days, mice were tested in an effortful operant task of motivation for sucrose reward (reward-to-effort valuation test, REV) using a progressive ratio schedule of reinforcement. Body weight of recipient mice was measured on each day of EV injection and behavioural testing. On the day after the last behavioural test, 4 days after the last EV injection, blood and spleen were collected for the determination of plasma cytokine/chemokine levels and mRNA-Seq, respectively.

Experiment 3 investigated the effects of NAc infusion of EVs isolated from plasma of LPS-challenged donor mice on physical status, reward-directed behaviour, and NAc tissue mRNome in recipient mice. A pilot study was conducted to assess the uptake of EV RNA by NAc cells. For this, donor-mice plasma EVs were isolated, and their RNA content labelled using a fluorescent dye. Recipient mice were implanted with a bilateral cannula projecting onto NAc in each hemisphere and, after recovery, were infused with RNA-labelled plasma EVs once per day for 3 days. Immunohistochemistry was then conducted to determine EV RNA uptake by different cell types in NAc tissue. In the main experiment, donor mice received LPS (1 mg/kg; *n* = 6) or SAL (*n* = 6), blood was collected after 5 h, and plasma EVs were isolated by precipitation and resuspended in artificial cerebrospinal fluid (aCSF). Recipient mice underwent stereotactic surgery for bilateral NAc cannulation. Following recovery, they received NAc infusion of LPS-EV-aCSF (*n* = 10), SAL-EV-aCSF (*n* = 10), or aCSF only (*n* = 10) on each of 3 days. For EV groups, each mouse was injected with EVs derived from the same plasma pool and with an estimated 1 × 10^9^ EVs per day. Previous studies reported increased markers of neuroinflammation after a single intracerebroventricular infusion of 3 × 10 ^8^–2 × 10^12^ EVs [11,27]. On the next 3 days, mice receiving LPS-EVs or SAL-EVs were tested in the REV task. Body weight of recipient mice was measured on each day of injection and behavioural testing. On the day after the last behavioural test and/or four days after the last EV/aCSF infusion, brains were collected, and NAc tissue samples were processed for mRNA-Seq. Analysis of differential gene expression followed by pathway over-representation analysis was conducted.

### 4.3. Lipopolysaccharide (LPS) Administration and Blood/Lymph Node Collection

Mice received a single intraperitoneal (i.p.) injection of LPS (*E. coli*, O127:B8, Sigma, St. Louis, MO, USA) at 1 mg/kg or physiological saline (SAL). Mice were sacrificed at 5 h post-LPS administration in the case of the LPS pilot miRNA and main miRNome Experiment 1 and the donor mice for Experiments 2 and 3. Mice were deeply anesthetized with a single i.p. injection of Pentobarbital-Natrium (100 mg/kg) and blood (500–800 µL) was collected via cardiac puncture into EDTA-coated vacutainers (S-Monovette 1.2 mL K3E, Sarstedt). Blood was then transferred to Protein LoBind tubes (Eppendorf) and centrifuged for 20 min at 2000× *g* and room temperature (RT). The resulting plasma was centrifuged at 10,000× *g* for 10 min at RT, then transferred to a fresh Protein LoBind tube and, in cases where EV isolation was not conducted immediately, snap-frozen on dry ice before storage at −80 °C. In Experiment 2, axillary and inguinal lymph nodes were also dissected at the time of blood collection. Lymph node tissue from all LPS and, separately, from all SAL donor mice, was pooled, and then homogenized on a cell-strainer tube equipped with a nylon mesh (Falcon) in 2 ml phosphate buffer saline (PBS). The homogenate was centrifuged at 500× *g* for 5 min and RT for cell-depletion; the cell-free supernatant was first centrifuged at 3000× *g* for 15 min and RT, then transferred to a fresh Protein LoBind tube, centrifuged at 10,000× *g* for 20 min and RT, and finally transferred to a fresh Protein LoBind tube before snap-freezing on dry ice and storage at −80 °C until EV isolation.

### 4.4. Chronic Social Stress (CSS)

CSS was conducted as described previously [25]. Briefly, CSS comprised 15 days of continuous exposure of C57BL/6J mice to dominant, ex-breeder male CD-1 mice. Each day, one C57BL/6J mouse was paired with a CD-1 mouse for 30–60 s of attack or a maximum of 10 min. Thereafter the two mice were separated by a transparent, perforated divider and maintained in sensory contact. Each C57BL/6J mouse was placed with a different CD-1 mouse each day. The lower incisor teeth of CD-1 mice were trimmed every third day to prevent bite wounding. Control mice (CON) were kept in littermate pairs and handled daily for 1 min. Blood collection for EV isolation took place 24 h after the last CSS day (day 16) and was conducted as described for the LPS procedures.

### 4.5. Extracellular Vesicle Isolation

Two methods of EV isolation, size exclusion chromatography and polymer-based precipitation, have relative advantages to each other depending on the specific research question. In this study, the method of SEC was used in the biomarker part of the study, because it ensures relatively high purity and minimises the amount of non-EV associated miRNAs isolated. Meanwhile the precipitation method was used for the isolation of plasma and lymph node EVs in the adoptive transfer experiments, because it allows for a relatively high yield of EVs and it is relatively rapid, features which were desirable for our experimental design.

In the LPS/CSS-EV microRNA-Sequencing (miRNA-Seq) main Experiment 1, EVs were isolated from one 200 µL plasma aliquot per subject using size exclusion chromatography (SEC) [68]. Samples were thawed at 37 °C for 4 min and briefly centrifuged to collect residual volumes. The supernatant (170 µL) was applied onto a SEC column (qEV single 35 nm, Izon Science) Christchurch, New Zealand and eluted by progressive addition of freshly filtered PBS, allowing for separation and collection of five distinct 200 µL fractions containing EVs. Per sample, EV-rich fractions were pooled and then ultra-filtrated to a volume of 200 µL using an Amicon Ultra-4 10K Centrifugal Filter Device (Merck, Kenilworth, NJ, USA), to which 600 µL lysis buffer (Norgen Biotek, Thorold, ON, Canada) containing 1% β-mercaptoethanol were added. Lysed samples were frozen immediately on dry ice and stored at −80 °C until RNA extraction. 

In the quantitative reverse transcription PCR (RT-qPCR) pilot Experiment 1, and from the donor mice in Experiments 2 and 3, EVs were isolated from plasma using the Total Exosome Isolation Kit from Plasma (Invitrogen, Waltham, MA, USA), according to the manufacturer’s instructions. In pilot Experiment 1, EV isolation occurred directly after blood collection from one plasma aliquot (170 µL) per subject. In Experiments 2 and 3, plasma samples from all LPS or SAL donor mice were each aggregated into three pools of 1.3 mL (Experiment 2) or 450 µL (Experiment 3), then frozen and stored at −80 °C. On each day of EV isolation, a plasma pool was thawed at 37 °C for 4 min before EV isolation. EV isolation from lymph samples (Experiment 2) was performed using the Total Exosome Isolation Kit from other Biofluids (Invitrogen) according to a customized protocol as follows: lymph pools were thawed at 37 °C for 4 min, then incubated with 0.5 volumes of precipitating agent for 1 h at 4 °C, and finally centrifuged at 3000× *g* for 30 min at 4 °C. In Experiments 1 and 2, isolated EVs were resuspended in physiological saline, whilst in Experiment 3 they were resuspended in artificial cerebrospinal fluid (aCSF: sodium chloride 1470 mM, potassium chloride 27 mM, calcium chloride dihydrate 12 mM, magnesium chloride hexahydrate 8.5 mM, and disodium hydrogen phosphate 10 mM, in HPLC grade water; pH 7.4). EV resuspension was achieved by shaking at 300 rpm and 37 °C for 30–60 min.

The presence of EVs in the purported EV-rich preparations, obtained using size exclusion chromatography or the Total Exosome isolation kits, was validated in accordance with the Guidelines of the International Society for Extracellular Vesicles [69]. For details see below.

#### 4.5.1. Validation of Plasma EV-Rich Fractions Obtained by Size Exclusion Chromatography

*Tunable resistive pulse sensing (TRPS)*. Measurements were carried out by Nanotechnology.life Laboratories, Loughborough University (Loughborough, UK). Samples were analyzed on a tunable resistive pulse sensing qNANO platform following a standardized protocol [70] (Figure S1A). Briefly, an NP100 membrane (Izon Science) was used, and the set-up calibrated using carboxylated particles (diameter = 110 nm). Size and concentration calibration were performed using CPN100 standards at a concentration of 1010 particles/mL. The EV concentration at each size category was determined using a two-points pressure method, with pressures ranging between 0.3–2 kPa. All samples were run in triplicate.

*Transmission electron microscopy (TEM).* The procedure was conducted at the Core Biotechnology Services Electron Microscopy Facility, University of Leicester (Leicester, UK). Isolated and resuspended EVs (5 µL) were applied onto a freshly glow discharged carbon film grid for 2 min, washed with distilled de-ionized water, and then air dried. Negative staining was achieved by double incubation with 5 µL 1% uranyl acetate. Samples were viewed on a JEOL JEM-1400 TEM with an accelerating voltage of 120 kV. Images were collected using an EMSIS Xarosa digital camera running Radius software (Figure S1B). 

*Western blotting of EV-specific proteins*. EVs isolated from plasma (Figure S1C) and resuspended in PBS were lysed in radioimmunoprecipitation assay (RIPA) buffer containing protease inhibitor (cOmplete Mini, Merck). A Pierce BCA Protein Assay (Thermo Fischer, Waltham, MA, USA) was used to assess total protein concentration. Lysates (6 µg) were resolved onto a 12% polyacrylamide precast gel (Criterion TGX, Bio-Rad, Hercules, CA, USA) using 1× sodium dodecyl sulfate (SDS) running buffer. Samples were then transferred onto a polyvinylidene difluoride (PVDF) membrane for 7 min at 25 V using the Trans-Blot Turbo Transfer system (Bio-Rad). Membranes were rinsed with PBS 1% Tween (PBS-T) and blocked with PBS-T containing 5% non-fat milk powder for 1 h and RT before overnight incubation with primary antibody at 4 °C. Primary antibodies against the following EV marker proteins [69] were used and at the dilutions indicated: Alix (1:5000, Abcam, Cambridge, UK) and CD9 (1:2000, Abcam). Membranes were then washed in PBS-T (3 × 20 min), incubated for 1 h at RT with the appropriate horseradish peroxidase-conjugated secondary antibody (1:1000, Abcam), and finally washed in PBS-T (3 × 20 min). Lastly, membranes were incubated with detection solvent (Clarity ECL Western Detection System, Bio-Rad) and images were acquired using a Chemidoc MP Imaging system (Bio-Rad, Hercules, CA, USA).

#### 4.5.2. Validation of Plasma and Lymph Node EV Preparations Obtained Using the Total Exosome Isolation Kit

*Nanoparticle tracking analysis (NTA)*. EV-rich pellets isolated from plasma (Figure S1D) or lymph nodes (Figure S1G) were diluted in filtered PBS to the optimal concentration for particle detection (30–60 particles/frame), and run on a NanoSight instrument (NS300, Malvern). For each sample, 5 videos each of 30 s were recorded and analyzed to determine mean particle size and concentration.

*Transmission electron microscopy (TEM).* Procedures were conducted at the Center for Microscopy and Image Analysis, University of Zurich (Zurich, Switzerland). For plasma EVs, formvar-carbon coated grids (Polysciences) were coated with 25 µg/mL poly-L-lysine (Sigma). Isolated EVs were post-fixed for 30 min in 0.4% (*w/v*) paraformaldehyde in phosphate buffer (100 mM, pH 7.2), followed by quenching of aldehyde groups in Tris-glycine solution (50 mM Tris, 50 mM glycine, pH 8.0). Negative staining was performed with 4% aqueous tungstosilicic acid (Merck), followed by positive contrasting with a 1:4 (*w/v*) solution of 1% aqueous methyl cellulose (Sigma) and 2% tungstosilicic acid. Grids were then air-dried and examined using an EM10 transmission electron microscope (Zeiss, Jena, Germany) operating at 60 kV (Figure S1E). For lymph node EVs, undiluted EV samples (10–30 µL) were applied onto a freshly glow discharged formvar-copper coated film grids and negative staining was achieved by single incubation with 1% uranyl acetate. Grids were then air dried and images acquired using a Talos L120C transmission electron microscope (Thermo Fisher Scientific) and Maps software (Thermo Fisher Scientific) (Figure S1H). 

*Western blotting of EV-specific proteins*. Conducted on plasma EVs (40 µg protein; Figure S1F) and lymph node EVs (25 µg protein: Figure S1I), as described for plasma EVs isolated using size exclusion chromatography.

### 4.6. RT-qPCR of Plasma Extracellular Vesicle miRNAs

In Experiment 1, following isolation of EVs from plasma using the Total Exosome Isolation Kit and resuspension in PBS, extracellular RNA not contained in EVs was degraded by treatment with proteinase K and RNAse according to a published protocol [71]. Briefly, samples were incubated with 50 mg/mL proteinase K (Sigma) for 10 min at 37 °C, followed by incubation with 5 mM phenulmethulsulfonyl fluoride (PMSF, Sigma) for 10 min at RT. Afterwards, samples were incubated with 100 mg/mL RNase A (Thermo Fisher Scientific) for 15 min at 37 °C and lastly with 2 U/uL RiboLock RNase Inhibitor (Thermo Fisher Scientific) for 10 min at 37 °C. Finally, a solution containing 700 µL of QIAzol lysis reagent (Qiagen Hilden, Germany) and 1 µL synthetic spike-in miRNA UniSp2 (RNA Spike-In Kit, Qiagen) was added to each sample before storage at −80 °C. Total RNA was then extracted using the miRNeasy Micro Kit (Qiagen) according to the manufacturer’s instructions. On-column DNase digestion was also performed to remove potential genomic DNA contamination. Per sample, 6 µL RNA were used for reverse transcription, conducted using the miRCURY LNA RT kit (Qiagen) according to the manufacturer’s instructions. Another synthetic spike-in miRNA, UniSp6 (RNA Spike-In Kit, Qiagen) was added to the solution to normalize any potential differences in reaction efficiency between samples. The cDNA samples were stored at −20 °C until further processing. The qPCR reaction was prepared using the miRCURY LNA SYBR PCR Kit and miRNA-specific primers (Qiagen), and run using a 7900HT Fast real-time PCR System (Thermofisher Scientific). Samples were run in triplicate, whilst the spike-ins Unisp2 and Unisp6 were run in duplicate and in each plate. Data were analysed using SDS v2.3 software (Limburg, Netherlands); baseline and threshold were adjusted manually for each run. For statistical analysis, normalized cycle threshold (Ct) values were used: normalized Ct values were calculated as the difference between the mean Ct of the target miRNA and the mean Ct of UniSp2 and UniSp6. The quantile normalization approach [72] was implemented to identify outliers.

### 4.7. Extracellular Vesicle miRNA-Sequencing and Bioinformatics

In main Experiment 1, following isolation of EVs from plasma using size exclusion chromatography and addition of lysis buffer, total EV RNA was extracted using the Plasma/Serum Purification Mini Kit (Norgen Biotek, Thorold, ON, Canada) according to the manufacturer’s instructions (‘Exosomal RNA Purification from Exosomes Already Purified via Ultracentrifugation, Exoquick, Filtration or any other Precipitation Method’). RNA concentration and size distribution were assessed using the Agilent RNA 6000 Pico kit and a 2100 Bioanalyzer system (Agilent Technologies, Santa Clara, CA, USA). Isolated RNA samples were stored at −80 °C until library preparation. Libraries for multiplexed sequencing were prepared using the QIAseq miRNA Library kit (Qiagen), according to the manufacturer’s instructions. Library quality was assessed using a TapeStation High Sensitivity DNA system (Agilent Biotechnologies). Individual sample libraries were then pooled using an equal amount of cDNA per sample, and miRNA-Seq was conducted on an Illumina HiSeq2500 System with a sequencing depth of 5 million reads per sample and sequencing configuration of 100 bp single-end.

The raw sequencing data were uploaded onto the GeneGlobe Data Analysis Center (Qiagen) for pre-processing and quantification. Firstly, 3′ adapters and low-quality bases were trimmed using Cutadapt. Next, the insert sequences and unique molecular identifiers (UMIs) were identified. Then, read mapping was performed according to a sequential alignment strategy using Bowtie: reads were mapped to miRBase Mature, miRBase Hairpin, Non-coding RNA, mRNA, and other RNAs, to identify perfect matches; mapping to a species-specific miRBase mature database was then conducted (1–2 mismatches tolerated), and all remaining unmapped sequences were aligned to the mouse genome (Genome Reference Consortium GRCm38) to identify possible novel miRNA molecules. All reads assigned to each specific miRNA were quantified and the associated UMIs aggregated to count unique molecules. To assess whether any systematic amplification bias was introduced during library preparation, Spearman’s rank-order correlation analysis of total read counts versus total UMIs was conducted: high correlations (rho = 0.99–1.0) demonstrated an absence of bias.

Downstream expression analyses were conducted in R. MiRNAs with less than 10 counts in ≥50% of the samples in one of the two groups being compared were considered to be at background noise level and discarded. Spearman’s rank-order correlation coefficients were calculated for each subject’s miRNA counts versus the group mean miRNA count, to assess within-group homogeneity and identify possible outliers; no outlier was identified. MiRNome differential expression analysis was implemented using the Bioconductor package *EdgeR*. Firstly, aggregate counts were normalized to the trimmed mean of the M values (TMM). Differential expression analysis was then conducted using the quasi-likelihood F-test; nominal p values and adjusted p values were calculated, the latter using the Benjamini-Hochberg false discovery rate (FDR). Pair-wise differential expression analyses for LPS versus SAL and CSS versus CON were conducted. Threshold criteria for differential expression were *p* value < 0.01 and log_2_ fold change ≥1 or ≤−1.

Target-prediction and pathway analyses were performed with the data obtained in the differential expression analysis, using the database miRWalk v. 3.0 [73]. First, predicted targets of dysregulated miRNAs were identified: only interactions predicted by both the miRWalk and the miRDB database [74], and between miRNAs and the mRNA 3′ UTR, were considered. Standard enrichment analysis [75] on these predicted targets was then performed using miRWalk, and significantly enriched pathways (adjusted *p* value < 0.05 according to FDR) were retrieved from the database Kyoto Encyclopedia of Genes and Genomes (KEGG) and grouped into broader functional categories based on the KEGG classification. Functional categories including ≤2 pathways or ≤10% of the targets involved in all significantly enriched pathways were discarded; cancer-related pathways were also excluded from the analysis because of the strong database bias in favour of these pathways compared to other disease-related pathways. Target-prediction and pathway analyses were conducted separately for the following: the 15 most up-regulated miRNAs in LPS vs. SAL mice (ranked according to log_2_ fold change as adjusted and nominal *p* values were <10 × 10^−16^ for all 15 miRNAs), all (6) up-regulated miRNAs and the 15 most down-regulated miRNAs in CSS vs. CON mice (ranked based on p values), and the 10 miRNAs that were both up-regulated in LPS vs. SAL and down-regulated in CSS vs. CON mice.

### 4.8. Behavioural Training and Testing

In Experiments 2 and 3, the behavioural test procedures including preparation and training were carried out using a published protocol [76].

#### 4.8.1. Food Restriction Protocol

Individual body weight and weight of food eaten per pair of littermates were measured for five consecutive days and used to calculate mean 100% baseline values per mouse. Two days before the start of operant training mice were reduced to 90–95% baseline bodyweight (BBW) by food restriction and maintained within this range during training. Mice received some sucrose-coated pellets (20 mg, Dustless Precision Pellets, Bio-Serv, Flemington, NJ, USA) in the home cage to familiarize them with the operant reward. During training, mice were weighed prior to the session and were fed 1 h after the session. Upon completion of training, mice were brought up to 95–100% BBW by providing them with sufficient food based on their baseline food consumption and maintained within this range throughout the experiment including behavioural testing.

#### 4.8.2. Operant Training

Mice were trained in an operant task with sucrose reinforcement over 15 days. Operant training and testing were conducted in a purpose-built apparatus comprising 2 chambers (TSE Systems, Bad Homburg, Germany) [76]. Each chamber was equipped with a retractable lever, a feeder port for pellet delivery that could be closed with an automatic sliding door, and a pellet dispenser. Mice were placed alone in a chamber and learned to press the retractable lever positioned directly next to a feeder port on a lateral wall of the chamber; one press (fixed ratio 1) resulted in opening of the feeder port door and delivery of a sucrose-coated pellet. Simultaneously, the lever retracted and remained so until the mouse had collected the pellet and a 5 s timeout had elapsed. As a second training step, the operant lever was relocated to the rear wall of the chamber, so that mice now needed to move between the lever and the feeder port; the reinforcement schedule was again FR1. Completion of training was defined as 30 trials completed in each of 2 sessions. Once all mice were trained, they were presented with preferred chocolate-coated sucrose pellets (20 mg, Dustless Precision Pellets, Bio-Serv) in the home cage [34] and given one FR1 session with this reinforcement, which was also used in all test sessions.

#### 4.8.3. Reward-to-Effort Valuation (REV) Test

As completion of the operant training, mice were given a reward-to-effort valuation (REV) test which deployed a progressive ratio schedule (PRS) of reinforcement, i.e., operant effort required for successive rewards increase across trials. The motivation scores in this baseline REV test were used to counter-balance mouse allocation to experimental groups. In Experiment 2, the PRS used was 5 trials at FR1 (FR1 × 5) followed by increments of 2 i.e., FR1 × 5, FR3 × 5, FR5 × 5 and so on, whilst in Experiment 3 increments of 4 were used i.e FR1 × 5, FR5 × 5, FR9 × 5 and so on. The total session duration was 30 min and no break point was used. Measures of interest were total number of lever presses, total number of pellets earned and, final ratio attained. For the behavioural tests of EV effects in Experiments 2 and 3, mice were given daily REV tests on three consecutive days, using a session duration of 45 min.

### 4.9. Intravenous Administration of Plasma-Lymph Node EVs to Recipient Mice

#### 4.9.1. Pilot Study

To determine whether exogenous plasma EVs administered intravenously would access the brain, blood was collected and plasma EVs isolated from naïve donor mice using the Total Exosome Isolation Kit from plasma. Following isolation, EVs were labelled using a non-lipophilic EV-specific fluorescent dye (ExoGlow^TM^-Vivo EV Labeling Kit, Near IR; System Biosciences; excitation/emission: 784/806 nm, Palo Alto, CA, USA) according to the manufacturer’s instructions, and then resuspended in physiological saline. Recipient mice were administered 0.5 mg/kg LPS i.p. at 16–18 h prior to intravenous EV infusion with the aim to establish low-level inflammatory conditions expected to increase blood brain barrier (BBB) permeability [77], thereby potentially facilitating EV transfer across the BBB. On the day of infusion, mice were placed into a Plexiglas restrainer (Indulab, Medellin, Colombia) and their tail immersed into water at 42 °C for 30 s to dilate the tail veins. The tail was then quickly dried with a cloth, and approximately 150 µL of labelled EVs, and non-labelled physiological saline in the case of control mice, were injected into one of the lateral veins using an insulin syringe (insulin U-100 0.5 mL micro-fine, 29G, Becton Dickinson). When mice were injected with an equivalent volume of ExoGlow-labelled saline, this distributed readily in the body and brain; the EV isolation from plasma was not 100% efficient such that ExoGlow-labelled EV-depleted plasma also displayed a signal. Therefore, non-labelled saline was used as the negative control. After injection, the site was cleaned, and the mouse returned to its cage. Imaging was conducted using the IVIS Lumina XR System (Perkin Elmer, Waltham, MA, USA) starting at 1 h and repeated at 2, 4, and 6 h, post-injection. Mice were anesthetized inside an induction chamber using 2% isoflurane (in pure oxygen), shaved on the cranium, abdomen, thorax and throat, and then placed inside the imaging apparatus (alternatively on the abdomen and back) with their snout in a nose cone for delivery of 1.5% isoflurane in oxygen. Following in vivo imaging, mice were perfused transcardially with ice-cold PBS for 5 min, and then brain, spleen, and liver were collected. Dissected organs were kept on ice and imaged immediately using the same settings used for the in vivo image acquisition. Images were acquired using the following combinations of excitation/emission filters: 710/760 nm, 710/780 nm, 710/800 nm, 745/800 nm, 745/820 nm, and 745/840 nm. Images were analysed using the Living Image software (v. 4.7.3; Perkin Elmer), and the Spectral Unmixing method was applied to subtract tissue auto-fluorescence from the ExoGlow-specific signal [78]. In both in vivo and ex vivo images, fluorescent ExoGlow-specific signal was visible in those mice infused with ExoGlow EVs but not in those infused with unlabelled saline (Figure S5).

#### 4.9.2. Main Study

In the main Experiment 2, recipient mice were injected into the lateral tail vein with SAL only or with EVs isolated from plasma and lymph nodes of LPS-or SAL-treated donor mice using the Total Exosome Isolation kits (see Extracellular vesicle isolation). The plasma aliquots from LPS or SAL donor mice were pooled, separately (3.7 mL plasma per donor group) as were lymph node aliquots (7.2 mL lymph solution per donor group). Plasma and lymph node EVs from the two donor groups were resuspended in 2.7 mL physiological saline each, pooled, and further aliquoted and pooled in order to obtain three aliquots of 1.8 mL plasma-lymph node EV solution per donor group. Therefore, all LPS-EV recipient mice were injected with EVs derived from the same plasma-lymph node pool and all SAL-EV recipient mice were injected with EVs derived from the same plasma-lymph node pool. The EVs in these pools were isolated on the day prior to recipient mice injection and stored at 4 °C across the 3 days of injection, with one EVs pool being used per day on three successive days. One aliquot of 5 mL physiological saline was prepared for the SAL recipient mice and stored at 4 °C throughout the injection days. Recipient mice were administered with 0.5 mg/kg LPS i.p. at 16–18 h before the first EV/SAL injection. On injection days, mice were brought to the experimental room, weighed, and injected with 150 µL plasma/lymph node EV solution of an estimated 4 × 10 ^10^ particles (LPS-EV or SAL-EV) or SAL only.

### 4.10. Immunoassay of Plasma Cytokines and Chemokines

At the end of Experiment 3, mice were deeply anesthetized with a single injection of Pentobarbital-Natrium (100 mg/kg, i.p.) and blood was collected via cardiac puncture into EDTA-coated vacutainers (S-Monovette 1.2ml K3E, Sarstedt, Nimbrecht, Germany ). Blood was transferred to Protein LoBind tubes, centrifuged at 860× *g* for 15 min at 4 °C, and finally transferred to fresh Protein LoBind tubes and snap-frozen on dry ice before long-term storage at −80 °C. Plasma concentrations of 19 pro-inflammatory molecules were assessed using the electrochemiluminescence V-PLEX Mouse Cytokine 19-Plex Kit (Meso Scale Diagnostic LLC.) according to the manufacturer’s instructions. Namely, interleukin (IL)-1β, IL-2, IL-4, IL-5, IL-6, IL-9, IL-10, IL-12p70, IL-15, IL-17A/F, IL-30, IL-33, monocyte chemoattractant 1, macrophage inflammatory protein (MIP)-1α, MIP-2, tumor necrosis factor α, interferon γ, interferon γ-induced protein 10, and the chemokine KC/GRO. Concentrations were measured using a dedicated electrochemiluminescence reader (MESO QuickPlex SQ 120, Meso Scale Diagnostic LLC) and data were interpolated from standard curves and analysed using the Discovery Workbench v 4.0 software (Meso Scale Diagnostic LLC, Rockville, MD, USA).

### 4.11. RNA-Sequencing and Bioinformatics of Spleen Tissue Samples

In Experiment 2, after blood collection, recipient mice were perfused transcardially with ice-cold filtered PBS (pH 7.4) for 5 min. Spleens were dissected and immediately frozen on dry ice and stored at −80 °C. Frozen spleens were microdissected using a surgical scalpel and 20–25 mg tissue was used for RNA isolation. RNA was extracted using the RNEasy Mini kit (Qiagen) according to the manufacturer’s instructions, including removal of genomic DNA. RNA concentration and RNA quality number (RQN) were assessed using a Fragment Analyzer (Advanced Analytical Technologies, Inc.) according to the SS Total RNA 15nt method; RQN of RNA samples was 5.8–8.8. Poly-A enriched mRNA libraries were prepared using the TruSeq stranded mRNA protocol (Illumina), and library quality was assessed on a Fragment Analyzer (Advanced Analytical Technologies, Inc.). RNA-Sequencing (RNA-Seq) was performed on an Illumina NovaSeq 6000 system, with sequencing depth of 20 million reads per sample and sequencing configuration of single-end 100 bp.

Raw sequencing data were uploaded onto the data analysis platform Sushi [79]. Reads were quality-checked with FastQC, sequencing adapters were removed with fasta [80] and aligned to the mouse reference genome and transcriptome (GENCODE, GRCm38.p5, release 91) using STAR v2.6.1 [81]. Transcripts with less than 10 counts in ≥ 50% of the samples in one of the two groups being compared were considered to be at background noise level and discarded. Distribution of the reads across genomic isoform expression was quantified using the R package *Genomic Ranges* [82] from Bioconductor Version 3.10. Minimum mapping quality, as well as minimum feature overlaps, were set to 10; multi-overlaps were allowed. Differential expression analysis was conducted with the Bioconductor R package *EdgeR* [83]: aggregate counts were normalized to the trimmed mean of the M values (TMM) and differential expression analysis was conducted using a generalised linear model regression and the quasi-likelihood F-test. The following pair-wise differential expression analyses were conducted: LPS-EVs versus SAL-EVs, LPS-EVs versus SAL, and SAL-EVs versus SAL. Criteria thresholds for differential expression were *p* value < 0.01 and log_2_ fold change ≥1 or ≤−1.

### 4.12. Infusion of Plasma EVs into the Nucleus Accumbens (NAc)

#### 4.12.1. Stereotactic Surgery for Bilateral Cannulation of Nucleus Accumbens

In Experiment 3 pilot and main studies, (recipient) mice underwent stereotactic surgery for implantation of a bilateral guide cannula projecting onto the nucleus accumbens (NAc), conducted according to a published protocol [84] with minor modifications. An incision was made at the cranial midline, skin and connective tissue were pulled to the side, and two holes were drilled into the cranium for insertion of the bilateral stainless steel guide cannula (26 G, centre-to-centre distance: 2.6 mm, length below pedestal: 4.5 mm; Bilaney Consultants). The stereotactic coordinates used were bregma AP + 1.7, ML ± 1.3, and DV −3.2 (mm) [85]. As established in pilot surgeries, these coordinates ensured guide cannula projection immediately dorsal to and avoiding tissue damage in the NAc. Pilot infusions using fluorescent-labelled EVs demonstrated targeted EV delivery to a region spanning the NAc shell-core border. Stable adhesion of the guide cannula onto the cranium was achieved as previously described [84]. A stainless-steel dummy cannula (0.02 mm diameter) was inserted into the guide cannula to which a nylon plastic dust-cap was secured. Mice recovered in the home cage on a warming pad for 1–2 h. Both mice per cage pair were operated on the same day, and mice were monitored daily on post-operative days 1–7.

#### 4.12.2. Pilot Study

To investigate uptake of EV RNA by NAc cells, EVs were isolated from plasma of naïve donor mice using the Total Exosome Isolation Kit from Plasma (Invitrogen) and labelled with a cell-permeable, non-lipophilic, RNA-selective fluorescent dye (SYTO^TM^ RNASelect^TM^ Green Fluorescent Cell Stain; Invitrogen; excitation/emission: 490/530 nm) according to the manufacturer’s instructions, followed by resuspension in aCSF (70 µL). Cannulated mice were infused onto the NAc with either SYTO-labelled EVs, non-labelled EVs, or SYTO-labelled aCSF. Infusions were conducted with a two-syringe infusion/withdrawal pump (World Precision Instruments, Sarasota, FL, USA) fitted with two glass Hamilton syringes (10 µL, 22 s G needle). A double internal cannula (33 G; Bilaney Consultants) was mounted onto a vinyl double connector and the latter connected to the two syringes. Recipient mice were anesthetized (2% isoflurane in pure oxygen), then transferred to a warming pad and placed in a nose cone delivering 1% isoflurane in oxygen; the absence of pain reflex responses was verified before starting the infusion. Bilateral infusion of 500 nL solution onto the NAc was conducted at a rate of 100 nL/min. The double internal cannula was left in place for 4 min to allow focal diffusion and reduce reflux, and then slowly withdrawn. The dummy cannula and dust-cap were re-inserted, and mice returned to the home cage. Three infusions were conducted on consecutive days, and 24 h after the last infusion brains were collected and fresh-frozen on powdered dry ice before storage at −80 °C. Brains were cryo-sectioned coronally at 20 μm, sections mounted on glass slides (Superfrost Plus microscope slides, Thermofisher), rinsed 2× with PBS and 1× with distilled water, and air-dried before storage at −80 °C. In some sections, mounting medium (Fluoroshield, Sigma Aldrich, St. Louis, MO, USA) was added immediately after air-drying and tissue was visualized using an epifluorescence microscope (Axio Observer, Zeiss) to identify the localization of SYTO-EV RNA signal. Based on these observations, the appropriate frozen sections were selected for immunofluorescence staining. Slides were thawed for 5 min at RT, then dehydrated for 15 min using an ice-cold solution of ethanol and acetone (1:1), air dried for 1–2 min, and finally washed in PBS (3 × 10 min). Individual sections were circled using a liquid blocker pen (Super pap pen mini, Daido Sangyo Co., Tokyo, Japan), incubated inside a moist chamber with blocking solution (1:1 PBS:distilled water solution containing 0.2% Triton and 10% goat or donkey serum) for 1 h at RT, and then incubated with primary antibody overnight at 4 °C inside the moist chamber. Primary antibodies against the following brain cell type-specific marker proteins were used at the dilutions indicated: NeuN (1:100, Abcam), Olig2 (1:50, RD Systems), CD68 (1:100, Serotec), and GFAP (1:100, Abcam). Slides were then washed in PBS (3 × 10 min), incubated with the appropriate fluorescent secondary antibody (1:500, AlexaFluor, Invitrogen) for 30 min at RT inside a moist chamber, washed again in PBS (3 × 10 min), and finally rinsed in distilled water before air drying and addition of mounting medium (Fluoroshield, Sigma Aldrich). Slides were stored at 4 °C for maximum two days before image acquisition. Images were acquired using an inverted confocal microscope (CLSM Leica SP8 inverse, Leica) and analysed using Imaris Cell Imaging software.

#### 4.12.3. Main Study

On each of the 3 days of infusion, plasma EVs were isolated from one of the 3 aliquots (450 µL) of the donor EV pool using the Total Exosome Isolation kit from plasma (Invitrogen) and resuspended in aCSF (70 µL). Starting on day 8 post-surgery and using the method described above for the pilot study, recipient mice were infused bilaterally onto the NAc with 1 µL of LPS-EV-aCSF or SAL-EV-aCSF of an estimated 1 × 10^9^ particles, or with aCSF only, once per day on 3 consecutive days. Three different syringe-connector-cannula systems, one for each test preparation, were used. Mice were controlled at 15 min and 5 h after each infusion.

### 4.13. RNA-Seq and Bioinformatics of NAc Tissue Samples

At the end of Experiment 3, recipient mice were perfused transcardially with ice-cold filtered PBS (pH 7.4) for 5 min. Brains were dissected, snap-frozen on powdered dry ice and stored at −80 °C. NAc microdissection was conducted as previously described [26]. Briefly, frozen brains were sectioned coronally at 1 mm intervals using a stainless steel brain matrix (Plastics One); the NAc (bregma 1.3 to 0.3 ± 0.2 mm) was identified referring to a mouse brain atlas [85] and microdissected bilaterally using a brain punch (∅ = 1.0 mm). The tissue damage due to the guide cannulae was visible and allowed for accurate identification of the infused area of tissue. Bilateral biopsies per mouse were combined and placed in 350 μL lysis RLT buffer (Qiagen) containing 2% dithiothreitol 2M, and then homogenized using a tissue lyser (Mixer-Mill 300, Qiagen) with stainless steel beads (∅ = 5 mm, Retsch). Total RNA was isolated using the RNeasy Plus Micro Kit (Qiagen) according to the manufacturer’s instructions. RNA sample concentration and RNA integrity number (RIN) were assessed using a High Sensitivity RNA ScreenTape System (Agilent Biotechnologies); all samples had a RIN > 8.5, and the entire remaining amount was used for RNA-Seq. Poly-A enriched mRNA libraries were prepared according to the Smart Seq2 protocol (Illumina), and library quality was assessed using a TapeStation High Sensitivity DNA system (Agilent Biotechnologies). Using an equal amount of cDNA per sample library, pools of cDNA libraries were prepared, and RNA-Seq was conducted on an Illumina NovaSeq 6000 System with a sequencing depth of 20 million reads per sample and sequencing configuration of 100 bp single-end. Raw sequencing data were processed, and differential gene expression analysis was conducted as described for the spleen RNA-Seq. The following pair-wise differential gene expression comparisons were performed: LPS-EV-aCSF versus SAL-EV-aCSF, LPS-EV-aCSF versus aCSF, and SAL-EV-aCSF versus aCSF. Criteria thresholds for differential expression were *p* value < 0.01 and log_2_ fold change ≥1 or ≤−1. Over-representation analysis of the ten genes significantly up-regulated in LPS-EV-aCSF mice compared to both SAL-EV-aCSF and aCSF mice was performed using the Molecular Signatures Database v7.4 [75,86], and significantly enriched pathways (adjusted *p* < 0.05 using FDR) were retrieved from the Gene Ontology-Biological Processes database.

### 4.14. Statistical Analysis

Statistical analysis was conducted using GraphPad Prism 7 and SPSS v25. Body weight and behavioural analyses (Experiments 2 and 3) were performed using a two-way mixed-model repeated measure analysis of variance (ANOVA), with significant within-subject main effects analysed post hoc using Tukey’s test and between-subject main effects analysed post hoc using Fisher’s least significant difference (LSD) test. The behavioural data were analysed further using linear regression. Two-tailed unpaired Student’s *t*-test was used for comparisons of two independent groups. Data are expressed as mean ± standard error of the mean (SEM) except where linear regression was used, when the 95% confidence intervals are also given. Statistical significance was set at *p* ≤ 0.05.

## Figures and Tables

**Table 1:** Pathway analysis for the NAc genes upregulated in LPS-EV-aCSF mice compared with SAL-EV-aCSF and aCSF mice, depicted in Figure 6 ^1^.

GO-BP Pathways	*Cd14*	*Chil1*	*Hmox1*	*Ccl5*	*Ptx3*	*Cxcl1*	*Aoah*	*Clec4n*	FDR
Inflammatory response									0.0000003
Defence response									0.000001
Myeloid leukocyte activation									0.0005
Exocytosis									0.002
Positive regulation of extracellular signal transduction									0.002
Response to cytokine									0.004
Myeloid leukocyte-mediated immunity									0.01
Cell activation									0.01
Secretion									0.01
Response to biotic stimulus									0.01
Cell activation involved in immune response									0.01
Cellular response to molecule of bacterial origin									0.01
Positive regulation of signalling									0.01
Cellular response to biotic stimulus									0.01
Regulation of intracellular signal transduction									0.01
Cytokine production									0.01
Leukocyte-mediated immunity									0.01
KappaB kinase NF-KappaB signalling									0.01
Response to tumor necrosis factor									0.02
Innate immune response									0.02
Response to molecule of bacterial origin									0.02
Positive regulation of gene expression									0.02
Response to fungus									0.02
Lipopolysaccharide-mediated signalling pathway									0.03
Modulation by host of symbiont process									0.03
Defence response to other organisms									0.03
Positive regulation of cytokine production									0.03
Immune effector process									0.04
Positive regulation of multicellular organismal processes									0.05
Leukocyte migration									0.05
Regulation of vesicle-mediated transport									0.05
Negative regulation of viral process									0.05
Biological process involved in interaction with symbiont									0.05

^1^ Upregulated gene SAA3 did not map onto any entry of the Molecular Signature Database. Upregulated gene ADGRE1 did not contribute to any significantly over-represented pathway. Filled cells denote that the gene is associated with the GO-BP pathways indicated.

## Data Availability

The data presented in this study are available on request from the corresponding author.

## Supplementary Materials

The following supporting information can be downloaded at: https://www.mdpi.com/article/10.3390/ijms23031028/s1.
