# Supplementary material for: Evidence for Effects of Extracellular Vesicles on Physical, Inflammatory, Transcriptome and Reward Behaviour Status in Mice
Source: Int J Mol Sci. 2022 Jan 18;23(3):1028. doi: 10.3390/ijms23031028 (PMC8835024; doi:10.3390/ijms23031028)
Supplement: Supplementary file 1 [file ijms-23-01028-s001.zip › ijms-1443284-supplementary.pdf]

International Journal of
Molecular Sciences

Supplementary Materials: Figures and Tables

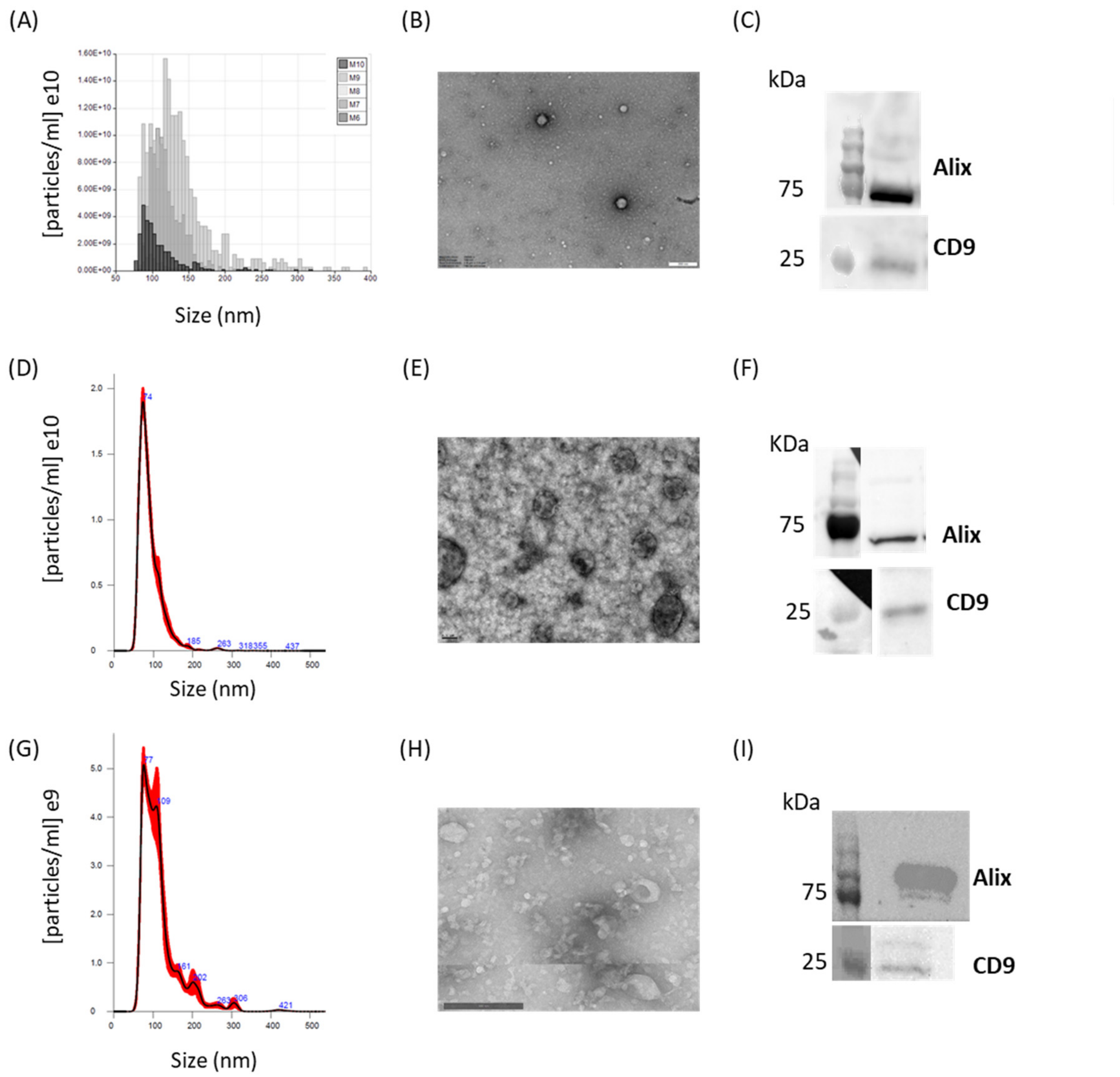

Figure S1. Experiment 1: Plasma EV total RNA concentrations. (A) Mice injected with LPS or SAL. (B) Mice that underwent CSS or CON handling. Individual values and mean ± standard error of the mean are given. ** $p < 0.01$, unpaired two-tailed Student's t-test.

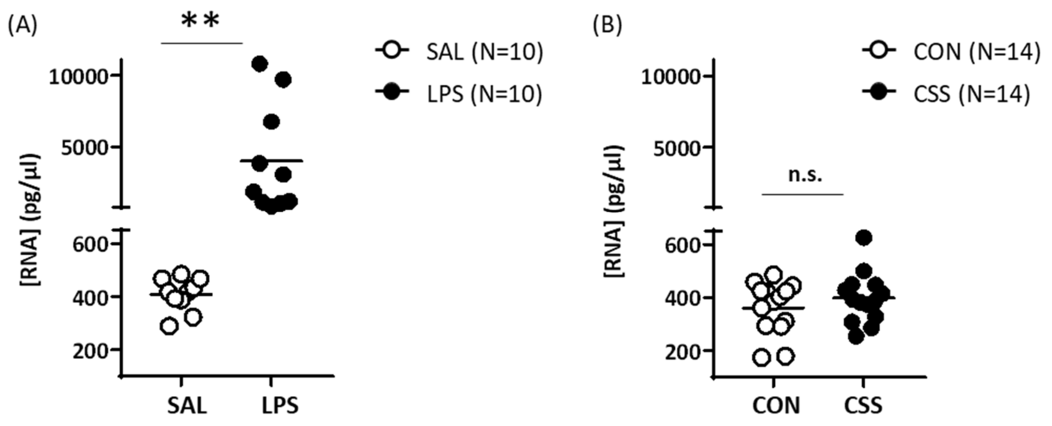

Figure S2. Experiment 1: Plasma EV total RNA concentrations. (A) Mice injected with LPS or SAL. (B) Mice that underwent CSS or CON handling. Individual values and mean ± standard error of the mean are given. ** $p < 0.01$, unpaired two-tailed Student's *t*-test.

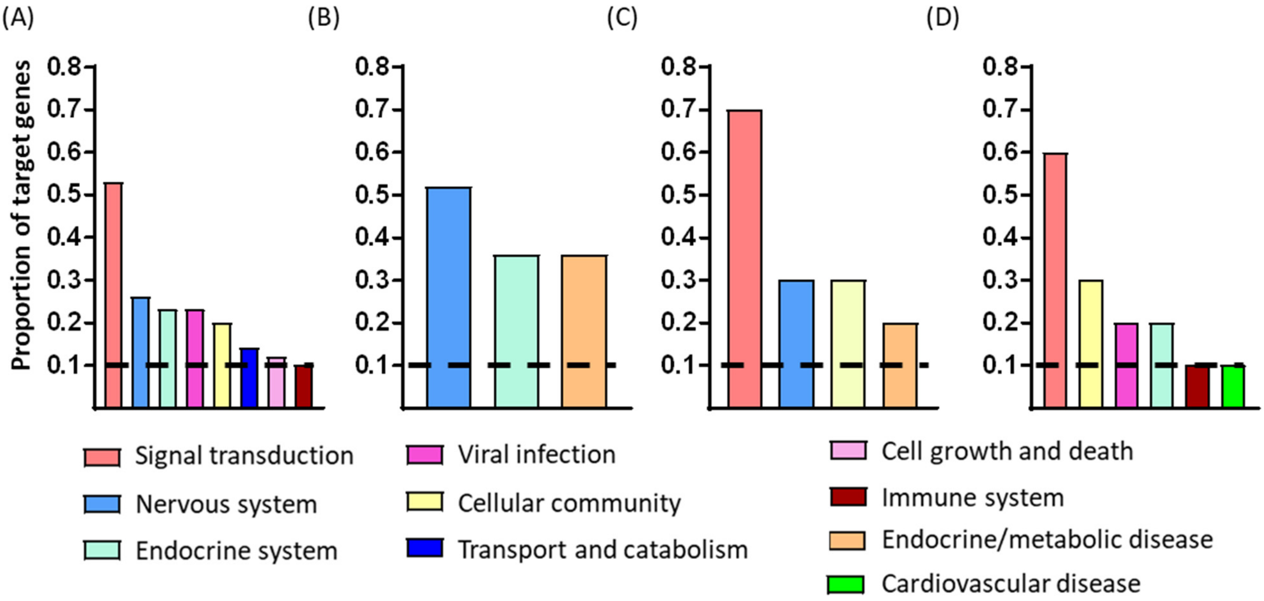

Figure S3**.** Experiment 1: Standard enrichment pathway analysis for the predicted targets of selected dysregulated miRNAs. (A) Pathways for the 15 most (based on p and $\log_2$ fold change values) up-regulated miRNAs in LPS- versus SAL-injected mice. (B) Pathways for the 6 up-regulated miRNAs in CSS- versus CON-exposed mice. (C) Pathways for the 15 most (based on p values) down-regulated miRNAs in CSS- versus CON-exposed mice. (D) Pathways for the ten miRNAs that were both up-regulated in LPS versus SAL mice and down-regulated in CSS versus CON mice. Standard enrichment pathway analysis was performed using miRWalk (v. 3.0) and significantly enriched pathways (BH-adjusted $p < 0.05$) were retrieved from the KEGG database and grouped into functional categories using

the KEGG classification system. Only functional categories including ≥ 2 enriched pathways and containing ≥ 0.1 of all target mRNAs were included; the dashed line indicates the 0.1 cut-off threshold.

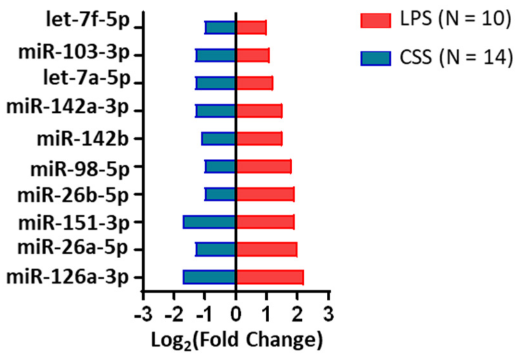

Figure S4. Experiment 1: Plasma EV miRNAs that were identified by miRNA-Seq to be both up-regulated in LPS versus SAL mice and down-regulated in CSS versus CON mice.

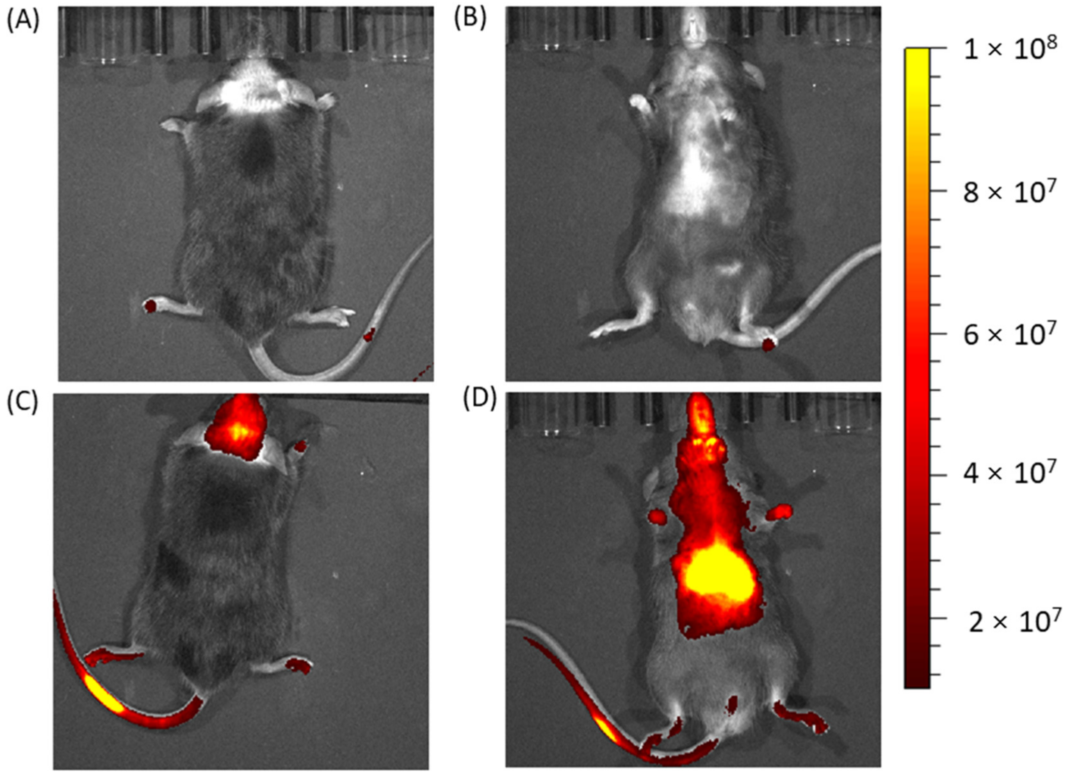

Figure S5. Experiment 2: Bio-distribution of exogenous plasma EVs injected intravenously. Experiment 2: (A-D) Representative whole-body *in vivo* images obtained with the IVIS Lumina XR System (Perkin Elmer) of mice at 2 h following tail vein injection. (A, B) Mouse injected with physiological saline only and imaged on the dorsal and ventral surface. (C, D) Mouse injected with EVs labelled with ExoGlow and imaged on the dorsal and ventral surface. Signal intensity depicts radiant efficiency ((p/sec/cm^2/sr)/(μW/cm^2)).

International Journal of
Molecular Sciences

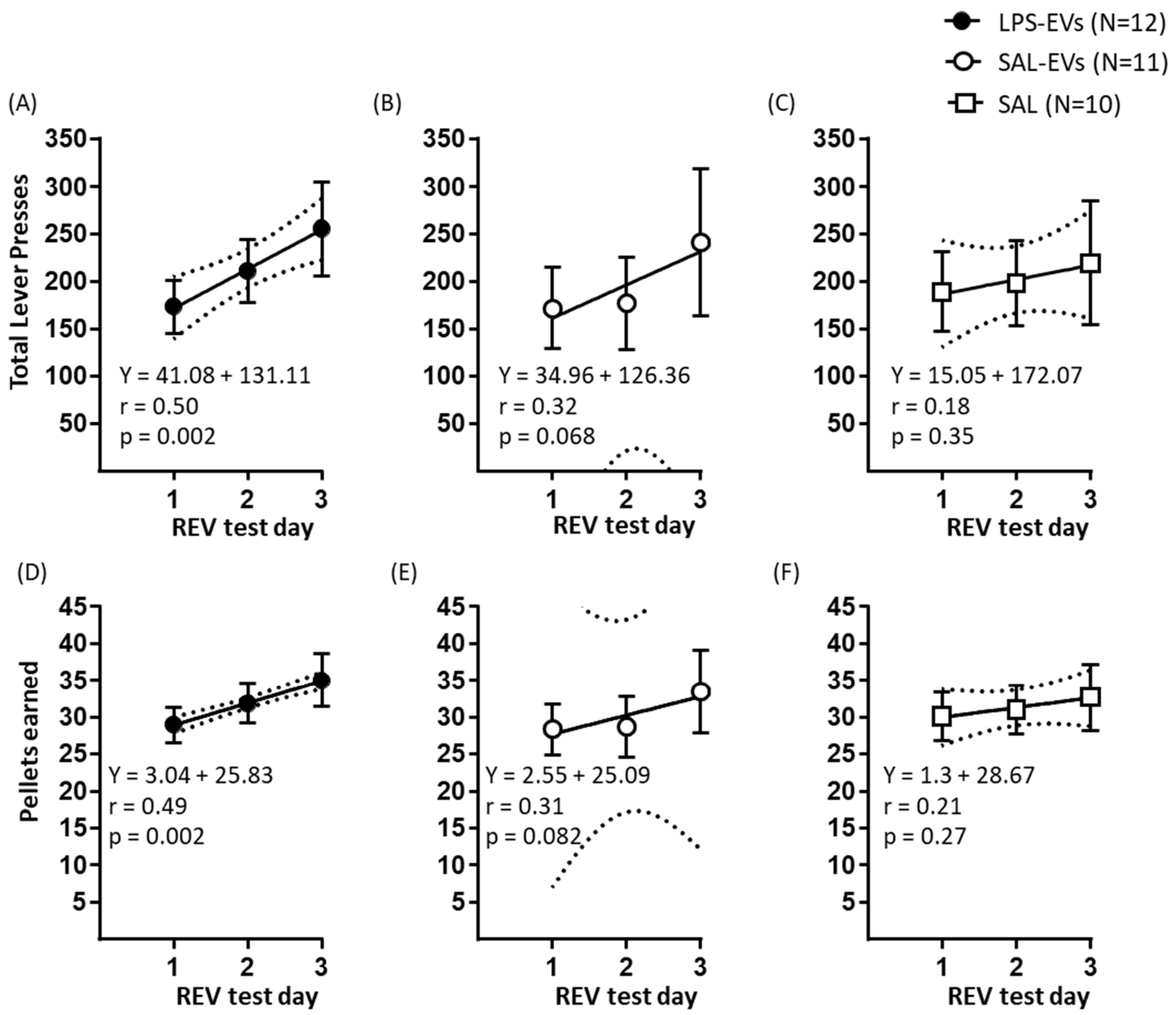

Figure S6. Experiment 2: Linear regression analysis for (A-C) Lever presses against test day and (D-F) Pellets earned against test day, in the REV test: (A, D) LPS-EV mice, (B, E) SAL-EV mice, (D, F) SAL mice. Data are expressed as mean ± 95% confidence interval; p values obtained from the ANOVA of the regression model. The solid line is for the regression equation and the dotted curves indicates the ± 95% confidence interval of the regression equation. For both measures of motivation, the regression was significant for LPS-EV mice specifically.

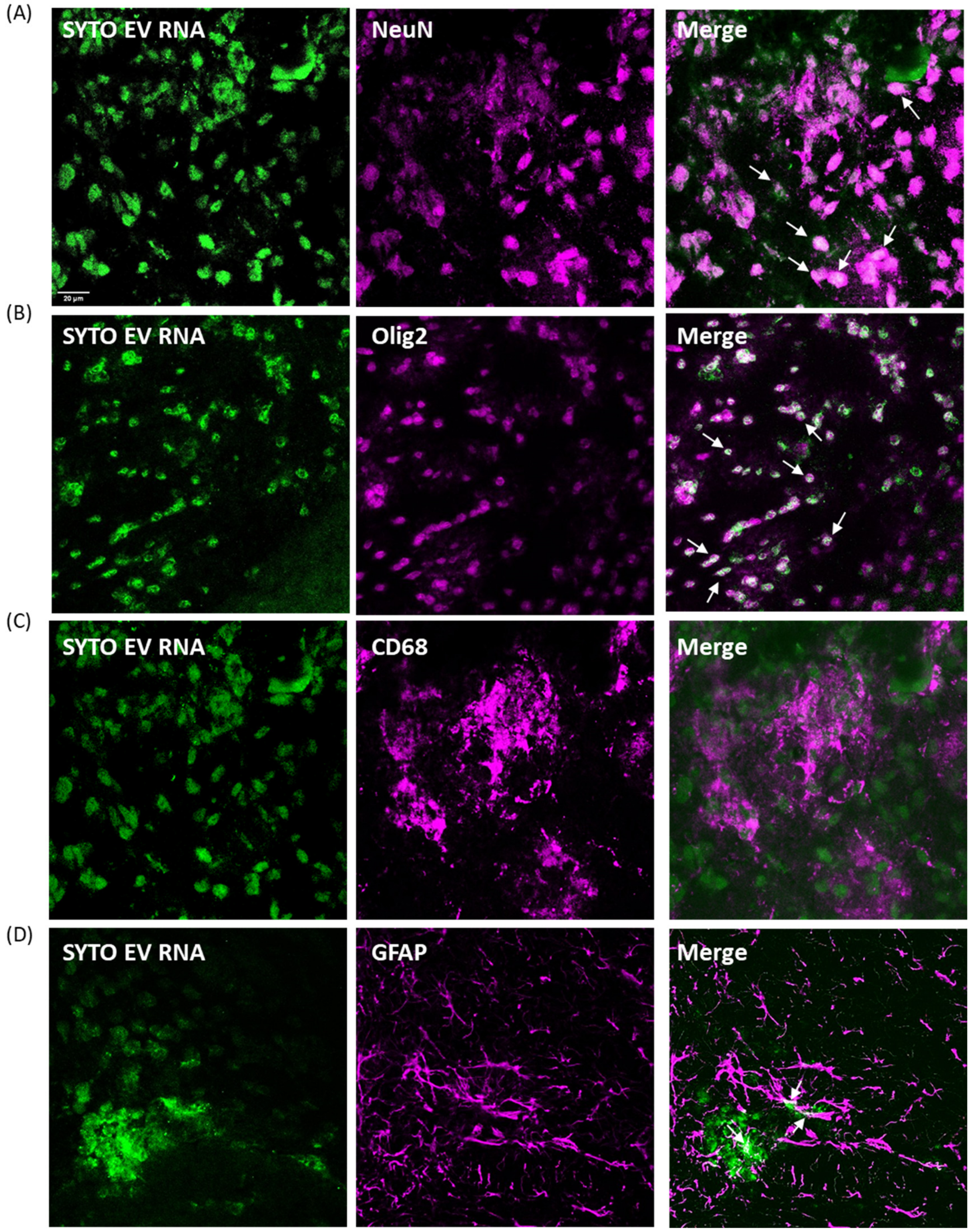

Figure S7. Experiment 3: Representative coronal brain images from mice injected *in vivo* in the NAc with EVs labelled with SYTO RNASelect fluorescent dye, followed by fresh brain collection, freezing and sectioning, and immunofluorescence with cell type-specific protein markers. For each cell type studied, the SYTO-stain image (green), immune-stain image (magenta), and their merged image, are shown. (A) NeuN for mature neurons. (B) Olig2 for mature oligodendrocytes. (C) CD68 for activated microglia. (D) GFAP for astrocytes. Examples of co-localization are indicated by arrows. Magnification 63×. Scale bar = 20 µm.

International Journal of
Molecular Sciences

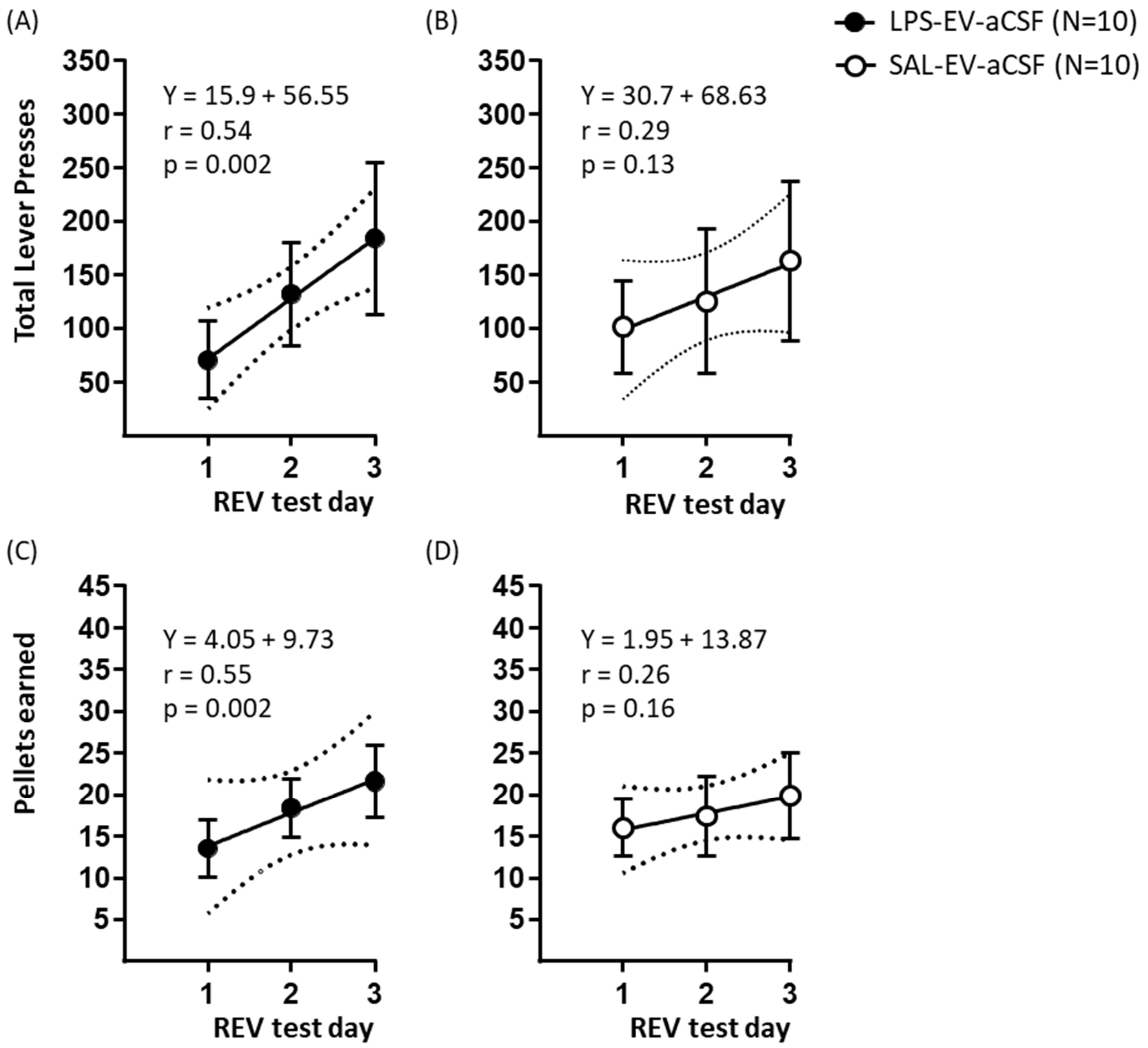

Figure S8. Experiment 3: Linear regression analysis for (A-B) Lever presses against test day and (C-D) Pellets earned against test day, in the REV test: (A, C) LPS-EV-aCSF mice, (B, D) SAL-EV-aCSF mice. Data are expressed as mean ± 95% confidence interval; p values obtained from the ANOVA of the regression model. The solid line is for the regression equation and the dotted curves indicates the ± 95% confidence interval of the regression equation. For both measures of motivation, the regression was significant for LPS-EV-aCSF mice specifically.

International Journal of *Molecular Sciences*

Table S1. Differentially expressed plasma EV miRNAs in LPS versus SAL mice

miRNA ID	SAL	LPS	FDR	p value	log_2FC
**Up-regulated**					
miR-144-5p	413	4228	<1.00E-16	<1.00E-16	3.3
miR-185-5p	325	1802	<1.00E-16	<1.00E-16	2.5
miR-144-3p	275	1352	<1.00E-16	<1.00E-16	2.3
miR-1839-5p	298	1438	<1.00E-16	<1.00E-16	2.3
let-7f-5p	9746	46436	<1.00E-16	<1.00E-16	2.2
miR-378a-3p	828	3814	<1.00E-16	<1.00E-16	2.2
miR-7a-5p	319	1466	<1.00E-16	<1.00E-16	2.2
miR-93-5p	2974	13401	<1.00E-16	<1.00E-16	2.2
let-7d-5p	1166	5228	<1.00E-16	<1.00E-16	2.2
let-7i-5p	10933	49058	<1.00E-16	<1.00E-16	2.2
miR-342-3p	7885	34957	<1.00E-16	<1.00E-16	2.1
miR-20a-5p	259	1147	<1.00E-16	<1.00E-16	2.1
miR-15b-5p	705	3110	<1.00E-16	<1.00E-16	2.1
miR-486b-5p	20868	92369	<1.00E-16	<1.00E-16	2.1
miR-148b-3p	327	1415	<1.00E-16	<1.00E-16	2.1
miR-146a-5p	8527	36889	<1.00E-16	<1.00E-16	2.1
miR-16-5p	91008	386799	<1.00E-16	<1.00E-16	2.1
miR-486a-5p	3865	16353	<1.00E-16	<1.00E-16	2.1
miR-451a	2192	9184	<1.00E-16	<1.00E-16	2.1
miR-146b-5p	453	1811	<1.00E-16	<1.00E-16	2.0
miR-25-3p	5180	20205	<1.00E-16	<1.00E-16	2.0
miR-103-3p	1295	5037	<1.00E-16	<1.00E-16	2.0
miR-486b-3p	407	1585	<1.00E-16	<1.00E-16	1.9
miR-106b-3p	314	1202	<1.00E-16	<1.00E-16	1.9
miR-34a-5p	205	780	<1.00E-16	<1.00E-16	1.9

miR-223-3p	11162	42373	<1.00E-16	<1.00E-16	1.9
miR-223-5p	269	1004	<1.00E-16	<1.00E-16	1.9
miR-486a-3p	521	1944	<1.00E-16	<1.00E-16	1.9
miR-92a-3p	6308	23323	<1.00E-16	<1.00E-16	1.9
miR-142a-3p	10255	37935	<1.00E-16	<1.00E-16	1.9
miR-191-5p	4467	16046	<1.00E-16	<1.00E-16	1.8
miR-744-5p	749	2663	<1.00E-16	<1.00E-16	1.8
miR-142b	361	1284	<1.00E-16	<1.00E-16	1.8
miR-351-5p	225	779	<1.00E-16	<1.00E-16	1.8
miR-16-2-3p	191	633	<1.00E-16	<1.00E-16	1.7
miR-328-3p	2204	7190	<1.00E-16	<1.00E-16	1.7
miR-19b-3p	304	967	<1.00E-16	<1.00E-16	1.7
miR-19a-3p	87	279	<1.00E-16	<1.00E-16	1.7
miR-425-5p	1879	5875	<1.00E-16	<1.00E-16	1.6
miR-15a-5p	410	1280	<1.00E-16	<1.00E-16	1.6
miR-186-5p	203	607	<1.00E-16	<1.00E-16	1.6
miR-22-3p	775	2283	<1.00E-16	<1.00E-16	1.6
miR-142a-5p	768	2273	<1.00E-16	<1.00E-16	1.6
miR-340-5p	251	729	<1.00E-16	<1.00E-16	1.5
miR-1198-5p	527	1498	<1.00E-16	<1.00E-16	1.5
miR-221-3p	1278	3598	<1.00E-16	<1.00E-16	1.5
miR-322-5p	462	1296	<1.00E-16	<1.00E-16	1.5
miR-423-3p	483	1344	<1.00E-16	<1.00E-16	1.5
miR-140-3p	3661	9642	<1.00E-16	<1.00E-16	1.4
miR-501-3p	517	1347	<1.00E-16	<1.00E-16	1.4
miR-361-5p	627	1584	<1.00E-16	<1.00E-16	1.3
miR-222-3p	245	615	<1.00E-16	<1.00E-16	1.3
miR-484	489	1215	<1.00E-16	<1.00E-16	1.3

International Journal of Molecular Sciences

MDPI

miR-93-3p	205	507	<1.00E-16	<1.00E-16		1.3
miR-298-5p	126	314	<1.00E-16	<1.00E-16		1.3
miR-1981-5p	216	519	<1.00E-16	<1.00E-16		1.3
miR-532-5p	371	886	<1.00E-16	<1.00E-16		1.3
miR-155-5p	313	720	<1.00E-16	<1.00E-16		1.2
miR-339-5p	251	569	<1.00E-16	<1.00E-16		1.2
miR-140-5p	219	500	<1.00E-16	<1.00E-16		1.2
miR-128-3p	1101	2404	<1.00E-16	<1.00E-16		1.1
miR-130b-3p	106	234	<1.00E-16	<1.00E-16		1.1
miR-421-3p	118	251	<1.00E-16	<1.00E-16		1.1
miR-7686-3p	120	726	0.02	0.00001	2.6	
miR-345-3p	180	593	0.02	0.00001	1.7	
mmu-let-7g-5p	1574	4603	0.02	0.00001		1.5
miR-101b-3p	1088	2979	0.02	0.00001	1.4	
miR-29b-3p	1099	2924	0.02	0.00001	1.4	
miR-30e-5p	3639	9412	0.02	0.00001	1.4	
miR-872-5p	185	399	0.02	0.00001	1.1	
miR-671-5p	150	316	0.02	0.00001	1.1	
miR-10a-3p	135	270	0.02	0.00001	1.0	
miR-423-5p	732	2333	0.03	0.00002	1.7	
miR-29a-3p	11541	31516	0.03	0.00002	1.4	
miR-28a-3p	149	352	0.03	0.00002	1.2	
miR-669a/o-3p	116	262	0.03	0.00002	1.2	
miR-674-3p	313	680	0.03	0.00002	1.1	
miR-361-3p	131	260	0.03	0.00002	1.0	
miR-7026-3p	93	731	0.05	0.00003	3.0	
miR-7087-5p	143	753	0.05	0.00003	2.4	
let-7a-5p	7207	28088	0.05	0.00003	1.9	

miR-7673-3p	199	1119	0.07	0.00004	2.5	
miR-378b	67	191	0.07	0.00004	1.5	
miR-664-5p	197	428	0.07	0.00004	1.1	
miR-107-3p	713	1557	0.07	0.00004	1.1	
miR-6935-3p	75	413	0.08	0.00005	2.4	
miR-215-3p	998	1941	0.1	0.00006	1.0	
miR-7236-3p	51	377	0.1	0.00007	2.8	
miR-26b-5p	3247	9041	0.1	0.00008	1.5	
miR-98-5p	122	361	0.2	0.00009	1.5	
miR-3068-5p	127	242	0.2	0.0001		1.0
miR-148a-3p	2116	5399	0.2	0.0001		1.4
miR-3100-5p	91	301	0.3	0.0002		1.7
miR-338-3p	81	196	0.3	0.0002		1.2
miR-883a-3p	77	273	0.4	0.0002		1.8
miR-30d-5p	18739	41296	0.4	0.0002		1.1
miR-10a-5p	4621	9228	0.6	0.0003		1.0
miR-7048-3p	173	377	0.6	0.0004		1.1
miR-8090	103	365	0.7	0.0004		1.8
miR-21a-5p	28066	70791	0.7	0.0004		1.3
miR-15b-3p	86	177	0.7	0.0005		1.1
miR-3970	75	184	0.8	0.0005		1.2
let-7c-5p	10886	22023	0.9	0.0005		1.0
miR-489-5p	28	141	1	0.0007		2.3
miR-32-5p	96	190	1	0.0007		1.0
miR-3547-3p	408	1190	1	0.0007		1.5
miR-7655-3p	327	877	1	0.0008		1.4
miR-126a-3p	22222	44268	1	0.0008		1.0
let-7a-1-3p/7c-2-3p	101	200	1	0.001		1.0

International Journal of Molecular Sciences

miR-547-5p	65	253	1	0.001	1.9
miR-26a-5p	6152	12880	1	0.001	1.1
miR-151-3p	577	1346	1	0.001	1.2
miR-471-3p	58	173	1	0.001	1.6
miR-3099-5p	116	258	1	0.002	1.1
miR-380-5p	77	194	1	0.002	1.3
miR-7027-5p	80	205	1	0.002	1.3
miR-344c-3p	154	329	1	0.005	1.1
miR-8111	116	247	1	0.005	1.1
miR-367-5p	4	15	1	0.008	2.0
miR-3108-3p	90	178	1	0.01	1.0
miR-8117	222	431	1	0.01	1.0
miR-3969	4	13	1	0.01	1.7
**Down-regulated**					
miR-3569-3p	6772	3247	0.1	0.0001	-1.1
miR-344f-5p	157	72	0.5	0.0003	-1.1

Expression levels for SAL and LPS are given in mean counts per million (CPM) normalized to the TMM; p value: nominal p value; FDR: FDR: p values calculated using the false discovery rate (FDR) according to the Benjamini-Hochberg method; log_2FC: fold change in logarithmic base 2 scale for LPS versus SAL.

Table S2. Differentially expressed plasma EV miRNAs in CSS versus CON mice

miRNA ID	CON	CSS	FDR	p value	log_2FC
**Up-regulated**					
miR-34c-5p	73	483	0.02	0.000012.7	
miR-346-5p	83	201	1	0.0008	1.3
miR-184-3p	252	882	1	0.0008	1.8
miR-9-5p	70	141	1	0.002	1.0
miR-1b-5p	351	744	1	0.01	1.1
miR-129-1-3p	7	13	1	0.01	1.0
**Down-regulated**					
miR-122-5p	40789	6769	<1.00E-16	<1.00E-16	-2.6
miR-26a-5p	6941	2732	0.02	0.00001-1.3	
mmu-let-7f-5p	18324	5674	0.02	0.00001-1.7	
mmu-let-7a-5p	17605	5519	0.04	0.00002-1.7	
miR-26b-5p	4559	1820	0.05	0.000031.3	
miR-7055-5p	289	107	0.05	0.000031.5	
miR-501-5p	525	99	0.1	0.00006-2.4	
miR-103-3p	1767	700	0.1	0.00008-1.3	
mmu-let-7b-5p	17663	7966	0.2	0.00009-1.2	
miR-151-3p	789	319	0.2	0.00009-1.3	
miR-190a-5p	90	35	0.2	0.0001	-1.4
miR-126a-3p	27001	13829	0.2	0.0001	-1.0
miR-374b-5p	104	52	0.4	0.0002	-1.1
miR-142b	431	210	0.4	0.0002	-1.0
miR-142a-3p	12082	5871	0.6	0.0003	-1.0
miR-98-5p	236	115	1	0.0008	-1.1
miR-682	259	119	1	0.004	-1.1

miR-1198-3p	143	61	1	0.005	-1.2
miR-615-5p	337	166	1	0.009	-1.0
miR-701-5p	205	97	1	0.01	-1.1

Expression levels for CON and CSS are given in counts per million (CPM) normalized to the TMM; p value: nominal p value; FDR: FDR: p values calculated using the false discovery rate (FDR) according to the Benjamini-Hochberg method; log_2FC: fold change in logarithmic base 2 scale for CSS versus CON.

Table S3a. Differentially expressed genes in the spleen of LPS-EV mice versus SAL-EV mice.

Gene_ID	Gene_name	Type	Biotypes	log_2FC	p value	FDR
ENSMUSG00000095633	*Igkv4-58*	protein_coding	IG_V_gene	2.22	0.003	0.9
ENSMUSG00000076709	*Ighv1-47*	protein_coding	IG_V_gene	2.02	0.01	0.9
ENSMUSG00000030730	*Atp2a1*	protein_coding	protein_coding	1.91	0.003	0.9
ENSMUSG00000056328	*Myh1*	protein_coding	protein_coding	1.55	0.009	0.9
ENSMUSG00000095442	*Ighv1-4*	protein_coding	IG_V_gene	-1.09	0.01	0.9
ENSMUSG00000073125	*Xlr3b*	protein_coding	protein_coding	-1.10	0.006	0.9
ENSMUSG00000076538	*Igkv13-84*	protein_coding	IG_V_gene	-1.21	0.003	0.9
ENSMUSG00000076545	*Igkv4-72*	protein_coding	IG_V_gene	-1.22	0.01	0.9
ENSMUSG00000076646	*Ighv2-6-8*	protein_coding	IG_V_gene	-1.40	0.01	0.9
ENSMUSG00000091971	*Hspa1a*	protein_coding	protein_coding	-1.60	0.005	0.9
ENSMUSG00000090877	*Hspa1b*	protein_coding	protein_coding	-1.83	0.002	0.9
ENSMUSG00000076710	*Ighv1-49*	protein_coding	IG_V_gene	-2.33	0.001	0.9

Table S3b. Differentially expressed genes in the spleen of LPS-EV mice versus SAL mice

Gene_ID	Gene_name	Type	Biotypes	log_2FC	p value	FDR
ENSMUSG00000076550	*Igkv4-63*	protein_coding	IG_V_gene	3.1	0.000731	0.3
ENSMUSG00000096452	*Ighv1-77*	protein_coding	IG_V_gene	2.8	0.007871	0.4
ENSMUSG00000095416	*Ighv1-12*	protein_coding	IG_V_gene	2.7	0.000621	0.3
ENSMUSG00000076709	*Ighv1-47*	protein_coding	IG_V_gene	2.2	0.013637	0.4
ENSMUSG00000095633	*Igkv4-58*	protein_coding	IG_V_gene	2.2	0.00694	0.4
ENSMUSG00000096715	*Igkv3-4*	protein_coding	IG_V_gene	2.2	0.000251	0.2

ENSMUSG00000030730	*Atp2a1*	protein_coding	protein_coding	1.8	0.010645	0.4
ENSMUSG00000096833	*Igkv4-55*	protein_coding	IG_V_gene	1.8	0.005824	0.4
ENSMUSG00000095351	*Igkv3-2*	protein_coding	IG_V_gene	1.8	0.000408	0.2
ENSMUSG00000056328	*Myh1*	protein_coding	protein_coding	1.7	0.011491	0.4
ENSMUSG00000021614	*Vcan*	protein_coding	protein_coding	1.7	0.003096	0.4
ENSMUSG00000094689	*Ighv1-81*	protein_coding	IG_V_gene	1.6	0.004276	0.4
ENSMUSG00000076525	*Igkv1-99*	protein_coding	IG_V_gene	1.6	0.000705	0.3
ENSMUSG00000076666	*Ighv14-4*	protein_coding	IG_V_gene	1.5	0.011158	0.4
ENSMUSG00000076596	*Igkv3-10*	protein_coding	IG_V_gene	1.5	0.00332	0.4
ENSMUSG00000076612	*Ighg2c*	protein_coding	IG_C_gene	1.4	0.008518	0.4
ENSMUSG00000094652	*Ighv1-42*	protein_coding	IG_V_gene	1.4	7.46E-05	0.1
ENSMUSG00000096074	*Ighv1-72*	protein_coding	IG_V_gene	1.4	0.000205	0.2
ENSMUSG00000095682	*Igkv3-1*	protein_coding	IG_V_gene	1.3	0.009052	0.4
ENSMUSG00000094694	*Ighv1-9*	protein_coding	IG_V_gene	1.3	0.00489	0.4
ENSMUSG00000094075	*Ighv1-80*	protein_coding	IG_V_gene	1.3	0.003858	0.4
ENSMUSG00000104452	*Ighv8-8*	protein_coding	IG_V_gene	1.3	0.001429	0.3
ENSMUSG00000076564	*Igkv12-46*	protein_coding	IG_V_gene	1.3	0.001654	0.3
ENSMUSG00000027718	*Il21*	protein_coding	protein_coding	1.2	0.002832	0.4
ENSMUSG00000094102	*Ighv9-2*	protein_coding	IG_V_gene	1.2	0.008141	0.4
ENSMUSG00000094006	*Igkv4-59*	protein_coding	IG_V_gene	1.2	0.009693	0.4
ENSMUSG00000076695	*Ighv1-18*	protein_coding	IG_V_gene	1.2	0.000531	0.3
ENSMUSG00000094546	*Ighv1-26*	protein_coding	IG_V_gene	1.2	0.000967	0.3
ENSMUSG00000076613	*Ighg2b*	protein_coding	IG_C_gene	1.1	0.001409	0.3
ENSMUSG00000096020	*Ighv1-75*	protein_coding	IG_V_gene	1.1	0.007514	0.4
ENSMUSG00000076549	*Igkv4-68*	protein_coding	IG_V_gene	1.1	0.001389	0.3
ENSMUSG00000076555	*Igkv4-57-1*	protein_coding	IG_V_gene	1.1	0.008459	0.4
ENSMUSG00000104217	*Gm37988*	protein_coding	protein_coding	1.1	0.006868	0.4
ENSMUSG00000076672	*Ighv3-6*	protein_coding	IG_V_gene	1.0	0.000404	0.2
ENSMUSG00000094862	*Ighv1-56*	protein_coding	IG_V_gene	1.0	0.009834	0.4

ENSMUSG00000039109	*F13a1*	protein_coding	protein_coding	1.0	0.008919	0.4
ENSMUSG00000073125	*Xlr3b*	protein_coding	protein_coding	-1.0	0.009213	0.4
ENSMUSG00000076665	*Ighv7-1*	protein_coding	IG_V_gene	-1.1	0.006913	0.4
ENSMUSG00000102364	*Ighv8-5*	protein_coding	IG_V_gene	-1.2	0.005068	0.4
ENSMUSG00000090877	*Hspa1b*	protein_coding	protein_coding	-1.3	0.006953	0.4

International Journal of *Molecular Sciences*

MDPI

Table S3c. Differentially expressed genes in the spleen of SAL-EV mice versus SAL mice.

Gene_ID	Gene_name	Type	Biotypes	log_2FC	p value	FDR
ENSMUSG00000096833	*Igkv4-55*	protein_coding	IG_V_gene	2.8	6.15E-05	0.2
ENSMUSG00000076525	*Igkv1-99*	protein_coding	IG_V_gene	2.7	5.25E-05	0.2
ENSMUSG00000096452	*Ighv1-77*	protein_coding	IG_V_gene	2.6	0.000726	0.5
ENSMUSG00000095416	*Ighv1-12*	protein_coding	IG_V_gene	2.6	0.00041	0.4
ENSMUSG00000096715	*Igkv3-4*	protein_coding	IG_V_gene	2.5	0.000317	0.4
ENSMUSG00000076710	*Ighv1-49*	protein_coding	IG_V_gene	2.4	0.004378	0.9
ENSMUSG00000094075	*Ighv1-80*	protein_coding	IG_V_gene	2.2	0.000142	0.4
ENSMUSG00000031896	*Ctrl*	protein_coding	protein_coding	2.1	0.01101	1.0
ENSMUSG00000076695	*Ighv1-18*	protein_coding	IG_V_gene	2.1	0.000577	0.5
ENSMUSG00000094862	*Ighv1-56*	protein_coding	IG_V_gene	2.0	0.000226	0.4
ENSMUSG00000076550	*Igkv4-63*	protein_coding	IG_V_gene	2.0	0.000427	0.4
ENSMUSG00000076545	*Igkv4-72*	protein_coding	IG_V_gene	1.9	0.000572	0.5
ENSMUSG00000076646	*Ighv2-6-8*	protein_coding	IG_V_gene	1.9	0.005883	1.0
ENSMUSG00000076564	*Igkv12-46*	protein_coding	IG_V_gene	1.9	0.000378	0.4
ENSMUSG00000098814	*Igkv19-93*	protein_coding	IG_V_gene	1.6	0.000886	0.5
ENSMUSG00000076612	*Ighg2c*	protein_coding	IG_C_gene	1.6	0.005289	1.0
ENSMUSG00000095351	*Igkv3-2*	protein_coding	IG_V_gene	1.5	0.000648	0.5
ENSMUSG00000096499	*Ighv1-5*	protein_coding	IG_V_gene	1.5	0.007094	1.0
ENSMUSG00000094652	*Ighv1-42*	protein_coding	IG_V_gene	1.5	3.55E-05	0.2
ENSMUSG00000094319	*Igkv4-54*	protein_coding	IG_V_gene	1.5	0.00504	1.0
ENSMUSG00000076555	*Igkv4-57-1*	protein_coding	IG_V_gene	1.4	0.004054	0.9
ENSMUSG00000093894	*Ighv1-53*	protein_coding	IG_V_gene	1.4	0.001157	0.6
ENSMUSG00000076672	*Ighv3-6*	protein_coding	IG_V_gene	1.4	0.000159	0.4
ENSMUSG00000104452	*Ighv8-8*	protein_coding	IG_V_gene	1.4	0.000604	0.5
ENSMUSG00000095007	*Igkv12-41*	protein_coding	IG_V_gene	1.4	0.001764	0.6
ENSMUSG00000094088	*Ighv1-64*	protein_coding	IG_V_gene	1.4	0.001901	0.6
ENSMUSG00000094694	*Ighv1-9*	protein_coding	IG_V_gene	1.3	0.001883	0.6

ENSMUSG00000094117	*Igkv3-12*	protein_coding	IG_V_gene	1.3	0.000762	0.5
ENSMUSG00000094194	*Ighv5-16*	protein_coding	IG_V_gene	1.2	0.001151	0.6
ENSMUSG00000076549	*Igkv4-68*	protein_coding	IG_V_gene	1.1	0.000767	0.5
ENSMUSG00000076576	*Igkv6-32*	protein_coding	IG_V_gene	1.1	0.010264	1.0
ENSMUSG00000027718	*Il21*	protein_coding	protein_coding	1.1	0.001918	0.6
ENSMUSG00000095429	*Ighv5-12*	protein_coding	IG_V_gene	1.1	0.001601	0.6
ENSMUSG00000094546	*Ighv1-26*	protein_coding	IG_V_gene	1.1	0.000324	0.4
ENSMUSG00000095285	*Ighv5-9*	protein_coding	IG_V_gene	1.0	0.006783	1.0
ENSMUSG00000096074	*Ighv1-72*	protein_coding	IG_V_gene	1.0	0.00332	0.8
ENSMUSG00000076543	*Igkv4-74*	protein_coding	IG_V_gene	1.0	0.00507	1.0
ENSMUSG00000095519	*Ighv1-66*	protein_coding	IG_V_gene	1.0	0.00529	1.0

Table S4a. Differentially expressed genes in the NAc of LPS-EV-aCSF mice versus SAL-EV-aCSF mice

Gene_ID	Gene_name	Type	Biotypes	log_2FC	p value	FDR
ENSMUSG00000035042	*Ccl5*	protein_coding	protein_coding	2.1	4.12E-08	0.0006
ENSMUSG00000040026	*Saa3*	protein_coding	protein_coding	2.6	1.3E-07	0.0009
ENSMUSG00000021322	*Aoah*	protein_coding	protein_coding	1.1	1.87E-05	0.04
ENSMUSG00000038173	*Enpp6*	protein_coding	protein_coding	1.0	4.08E-05	0.07
ENSMUSG00000024610	*Cd74*	protein_coding	protein_coding	1.4	6.81E-05	0.09
ENSMUSG00000073409	*H2-Q6*	protein_coding	protein_coding	1.5	8.7E-05	0.1
ENSMUSG00000030214	*Plbd1*	protein_coding	protein_coding	1.1	0.0001	0.1
ENSMUSG00000031344	*Gabrq*	protein_coding	protein_coding	1.1	0.0001	0.1
ENSMUSG00000023349	*Clec4n*	protein_coding	protein_coding	1.5	0.0002	0.1
ENSMUSG00000073411	*H2-D1*	protein_coding	protein_coding	1.5	0.0002	0.1
ENSMUSG00000060586	*H2-Eb1*	protein_coding	protein_coding	1.4	0.0002	0.1
ENSMUSG00000051439	*Cd14*	protein_coding	protein_coding	1.0	0.0003	0.1
ENSMUSG00000055413	*H2-Q5*	protein_coding	polymorphic_pseudogene	1.4	0.0004	0.1
ENSMUSG00000035929	*H2-Q4*	protein_coding	protein_coding	1.7	0.0004	0.1
ENSMUSG00000060550	*H2-Q7*	protein_coding	protein_coding	1.7	0.0005	0.2
ENSMUSG00000061232	*H2-K1*	protein_coding	protein_coding	1.6	0.0005	0.2

ENSMUSG00000001128	*Cfp*	protein_coding	protein_coding	1.3	0.0006	0.2
ENSMUSG00000036594	*H2-Aa*	protein_coding	protein_coding	1.3	0.0008	0.2
ENSMUSG00000006522	*Itih3*	protein_coding	protein_coding	1.0	0.0008	0.2
ENSMUSG00000015340	*Cybb*	protein_coding	protein_coding	1.6	0.001	0.3
ENSMUSG00000035493	*Tgfbi*	protein_coding	protein_coding	1.2	0.001	0.3
ENSMUSG00000005413	*Hmox1*	protein_coding	protein_coding	2.6	0.001	0.3
ENSMUSG00000069515	*Lyz1*	protein_coding	protein_coding	1.5	0.002	0.3
ENSMUSG00000073421	*H2-Ab1*	protein_coding	protein_coding	1.3	0.002	0.3
ENSMUSG00000024679	*Ms4a6d*	protein_coding	protein_coding	1.2	0.002	0.3
ENSMUSG00000060802	*B2m*	protein_coding	protein_coding	1.2	0.002	0.3
ENSMUSG00000004730	*Adgre1*	protein_coding	protein_coding	1.0	0.002	0.3
ENSMUSG00000040829	*Zmynd15*	protein_coding	protein_coding	1.1	0.002	0.3
ENSMUSG00000019987	*Arg1*	protein_coding	protein_coding	1.8	0.002	0.3
ENSMUSG00000002944	*Cd36*	protein_coding	protein_coding	1.6	0.002	0.3
ENSMUSG00000064246	*Chil1*	protein_coding	protein_coding	1.0	0.002	0.3
ENSMUSG00000000982	*Ccl3*	protein_coding	protein_coding	1.0	0.002	0.3
ENSMUSG00000029380	*Cxcl1*	protein_coding	protein_coding	1.1	0.002	0.3
ENSMUSG00000031111	*Igsf1*	protein_coding	protein_coding	1.3	0.003	0.3
ENSMUSG00000030560	*Ctsc*	protein_coding	protein_coding	1.0	0.003	0.3
ENSMUSG00000073418	*C4b*	protein_coding	protein_coding	1.0	0.003	0.3
ENSMUSG00000018920	*Cxcl16*	protein_coding	protein_coding	1.1	0.003	0.3
ENSMUSG00000047798	*Cd300lf*	protein_coding	protein_coding	1.2	0.004	0.3
ENSMUSG00000029371	*Cxcl5*	protein_coding	protein_coding	1.2	0.004	0.3
ENSMUSG00000012428	*Steap4*	protein_coding	protein_coding	1.2	0.005	0.3
ENSMUSG00000001348	*Acp5*	protein_coding	protein_coding	1.4	0.005	0.3
ENSMUSG00000079419	*Ms4a6c*	protein_coding	protein_coding	1.0	0.006	0.3
ENSMUSG00000031980	*Agt*	protein_coding	protein_coding	1.2	0.007	0.3
ENSMUSG00000038508	*Gdf15*	protein_coding	protein_coding	1.1	0.007	0.3
ENSMUSG00000062382	*Ftl1-ps1*	protein_coding	protein_coding	1.0	0.008	0.3

ENSMUSG00000024164	*C3*	protein_coding	protein_coding	1.4	0.01	0.3
ENSMUSG00000038642	*Ctss*	protein_coding	protein_coding	1.0	0.01	0.3
ENSMUSG00000112023	*Lilr4b*	protein_coding	protein_coding	1.6	0.01	0.3
ENSMUSG00000030144	*Clec4d*	protein_coding	protein_coding	1.2	0.01	0.3
ENSMUSG00000024672	*Ms4a7*	protein_coding	protein_coding	1.2	0.01	0.3
ENSMUSG00000069516	*Lyz2*	protein_coding	protein_coding	2.0	0.01	0.3
ENSMUSG00000027832	*Ptx3*	protein_coding	protein_coding	1.4	0.01	0.4
ENSMUSG00000005087	*Cd44*	protein_coding	protein_coding	1.0	0.01	0.4
ENSMUSG00000090639	*Gm20425*	protein_coding	protein_coding	1.0	0.01	0.4
ENSMUSG00000032554	*Trf*	protein_coding	protein_coding	1.0	0.01	0.4
ENSMUSG00000079014	*Serpina3i*	protein_coding	protein_coding	1.0	0.01	0.4
ENSMUSG00000022548	*Apod*	protein_coding	protein_coding	1.1	0.01	0.4

Table S4b. Differentially expressed genes in the NAc of LPS-EV-aCSF mice versus aCSF mice

Gene_ID	Gene_name	Type	Biotypes	log_2FC	p value	FDR
ENSMUSG00000021322	*Aoah*	protein_coding	protein_coding	1.1	0.00003	0.2
ENSMUSG00000035042	*Ccl5*	protein_coding	protein_coding	1.9	0.00006	0.3
ENSMUSG00000023349	*Clec4n*	protein_coding	protein_coding	1.5	0.0004	0.5
ENSMUSG00000029380	*Cxcl1*	protein_coding	protein_coding	1.2	0.0008	0.5
ENSMUSG00000064246	*Chil1*	protein_coding	protein_coding	1.2	0.001	0.5
ENSMUSG00000005413	*Hmox1*	protein_coding	protein_coding	2.8	0.001	0.5
ENSMUSG00000051439	*Cd14*	protein_coding	protein_coding	1.0	0.003	0.5
ENSMUSG00000040026	*Saa3*	protein_coding	protein_coding	1.8	0.004	0.6
ENSMUSG00000052305	*Hbb-bs*	protein_coding	protein_coding	3.2	0.004	0.6
ENSMUSG00000004730	*Adgre1*	protein_coding	protein_coding	1.1	0.004	0.6
ENSMUSG00000028364	*Tnc*	protein_coding	protein_coding	1.3	0.008	0.6
ENSMUSG00000023031	*Cela1*	protein_coding	protein_coding	1.1	0.008	0.6
ENSMUSG00000027832	*Ptx3*	protein_coding	protein_coding	1.5	0.01	0.6
ENSMUSG00000050063	*Klk6*	protein_coding	protein_coding	1.8	0.01	0.6
ENSMUSG00000027074	*Slc43a3*	protein_coding	protein_coding	1.1	0.01	0.6

International Journal of *Molecular Sciences*

ENSMUSG00000035783	*Acta2*	protein_coding	protein_coding	-1.6	0.007	0.6

International Journal of *Molecular Sciences*

Table S4c. Differentially expressed genes in the NAc of SAL-EV-aCSF mice versus aCSF mice

Gene_ID	Gene_name	Type	Biotypes	log_2FC	p value	FDR
ENSMUSG00000052305	*Hbb-bs*	protein_coding	protein_coding	4.7	0.001	0.7
ENSMUSG00000027514	*Zbp1*	protein_coding	protein_coding	-1.0	0.01	0.7
ENSMUSG00000029561	*Oasl2*	protein_coding	protein_coding	-1.0	0.01	0.7
ENSMUSG00000055413	*H2-Q5*	protein_coding	polymorphic_pseudogene	-1.2	0.01	0.7
ENSMUSG00000031111	*Igsf1*	protein_coding	protein_coding	-1.3	0.00	0.7
ENSMUSG00000031980	*Agt*	protein_coding	protein_coding	-1.6	0.01	0.7
ENSMUSG00000035783	*Acta2*	protein_coding	protein_coding	-1.7	0.01	0.7
